# YTHDF2 Is a Therapeutic Target for HCC by Suppressing Immune Evasion and Angiogenesis Through ETV5/PD‐L1/VEGFA Axis

**DOI:** 10.1002/advs.202307242

**Published:** 2024-01-21

**Authors:** Jingyuan Wen, Lin Xue, Yi Wei, Junnan Liang, Wenlong Jia, Tuying Yong, Liang Chu, Han Li, Shenqi Han, Jingyu Liao, Zeyu Chen, Yiyang Liu, Qiumeng Liu, Zeyang Ding, Huifang Liang, Lu Gan, Xiaoping Chen, Zhao Huang, Bixiang Zhang

**Affiliations:** ^1^ Hepatic Surgery Center, Tongji Hospital, Tongji Medical College Huazhong University of Science and Technology Wuhan 430030 China; ^2^ Clinical Medical Research Center of Hepatic Surgery at Hubei Province Wuhan 430030 China; ^3^ Hubei Key Laboratory of Hepato‐Pancreatic‐Biliary Diseases, Tongji Hospital, Tongji Medical College Huazhong University of Science and Technology Wuhan 430030 China; ^4^ National Engineering Research Center for Nanomedicine College of Life Science and Technology Huazhong University of Science and Technology Wuhan 430074 China; ^5^ Key Laboratory of Organ Transplantation, Ministry of Education; Key Laboratory of Organ Transplantation, National Health Commission; Key Laboratory of Organ Transplantation Chinese Academy of Medical Science Wuhan 430030 China

**Keywords:** angiogenesis, eIF3b, ETV5, immune evasion, Liver cancer, N^6^‐methyladenosine, targeted therapy, translation, YTHDF2

## Abstract

N6‐methyladenosine (m^6^A) modification orchestrates cancer formation and progression by affecting the tumor microenvironment (TME). For hepatocellular carcinoma (HCC), immune evasion and angiogenesis are characteristic features of its TME. The role of YTH N6‐methyladenosine RNA binding protein 2 (YTHDF2), as an m^6^A reader, in regulating HCC TME are not fully understood. Herein, it is discovered that trimethylated histone H3 lysine 4 and H3 lysine 27 acetylation modification in the promoter region of YTHDF2 enhanced its expression in HCC, and upregulated YTHDF2 in HCC predicted a worse prognosis. Animal experiments demonstrated that *Ythdf2* depletion inhibited spontaneous HCC formation, while its overexpression promoted xenografted HCC progression. Mechanistically, YTHDF2 recognized the m^6^A modification in the 5′‐untranslational region of ETS variant transcription factor 5 (ETV5) mRNA and recruited eukaryotic translation initiation factor 3 subunit B to facilitate its translation. Elevated ETV5 expression induced the transcription of programmed death ligand‐1 and vascular endothelial growth factor A, thereby promoting HCC immune evasion and angiogenesis. Targeting YTHDF2 via small interference RNA‐containing aptamer/liposomes successfully both inhibited HCC immune evasion and angiogenesis. Together, this findings reveal the potential application of YTHDF2 in HCC prognosis and targeted treatment.

## Graphical abstract

YTHDF2 acted as a tumor promoter by upregulating PD‐L1 and VEGFA expression in HCC. YTHDF2 recruited eIF3b to facilitate ETV5 mRNA translation, thereby enhancing transcriptional processes of PD‐L1 and VEGFA. From the clinical viewpoint, targeting YTHDF2 can concurrently inhibit PD‐L1 and VEGFA to suppress immune evasion and angiogenesis in HCC, which potentiates its translational value as a therapeutic target.

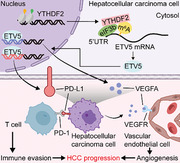

## 1 Introduction

Hepatocellular carcinoma (HCC) is the third leading cause of cancer‐associated death worldwide. HCC patients are often diagnosed at advanced stages and cannot undergo curative surgery.^[^ ^1^ ^]^ In previous decades, systematic therapies, including tyrosine kinase inhibitors, antiangiogenic antibodies and immune checkpoint inhibitors, have been developed to prolong the survival time of HCC patients.^[^ ^2^ ^]^ However, the prognosis of HCC is far from satisfactory, and elucidating the molecular mechanism underlying HCC formation and progression, as well as developing new effective targets for HCC treatment, remain challenging.

N^6^‐methyladenosine (m^6^A), which is the most prevalent internal modification of eukaryotic mRNAs, widely participates in mRNA splicing, nuclear export, stability and translation processes.^[^ ^3^ ^]^ M^6^A modifiers determine the fate of target RNAs and thereby influence protein expression, molecular pathways and cell phenotypes.^[^ ^4^ ^]^ Dysregulated m^6^A modifiers have been shown to function as oncoproteins or tumor suppressors with essential roles in cancer initiation, progression and therapy resistance;^[^ ^5^ ^]^ these findings highlight the therapeutic potential of targeting dysregulated m^6^A machinery to treat cancer. YTH N6‐methyladenosine RNA binding protein 2 (YTHDF2), which was the first discovered m^6^A reader,^[^ ^6^ ^]^ was found to degrade target mRNAs by recognizing m^6^A modifications and recruiting the CCR4‐NOT deadenylase complex through its N‐terminal 101–200 amino acid to initiate deadenylation.^[^ ^7^ ^]^ Additionally, YTHDF2 could also bind to m^6^A‐modified mRNAs in an HRSP12‐dependent manner, resulting in cleavage by RNase P/MRP (endoribonucleases).^[^ ^8^ ^]^ At the same time, some researchers reported that YTHDF2 stabilized mRNA transcripts and facilitated their translation,^[^ ^9^, ^10^, ^11^, ^12^ ^]^ but the exact molecular mechanisms are unclear. Targeting YTHDF2 can serve as a strategy to suppress cancer and enhance the efficacy of anti‐tumor treatments.^[^ ^13^, ^14^ ^]^ Nevertheless, the reported roles of YTHDF2 in HCC are inconsistent and even contradictory,^[^ ^10^, ^12^, ^15^, ^16^ ^]^ and the therapeutic value of YTHDF2 in HCC treatment remains underexplored.

Recent studies illustrate that, T cell‐mediated immune surveillance and vascular endothelial cell‐mediated angiogenesis are important components of the TME that promote the occurrence and development of HCC.^[^ ^17^, ^18^ ^]^ Immune checkpoint blockade by targeting PD‐L1/PD‐1 signaling has been considered a breakthrough for advanced HCC treatment.^[^ ^19^ ^]^ Multiple agents that target the vascular endothelial growth factor (VEGF) pathway, such as sorafenib, lenvatinib, and bevacizumab, have been evaluated and have shown clinical efficacy for the treatment of HCC.^[^ ^20^ ^]^ Of note, combined therapies (anti‐angiogenic therapy plus immune checkpoint inhibitor) have shown encouraging progress compared with single therapy.^[^ ^2^, ^21^ ^]^ In the clinic, the combination of atezolizumab (anti‐PD‐L1) and bevacizumab (anti‐VEGF) was approved as a first‐line treatment for advanced HCC in 2020 due to its outstanding antitumor effects.^[^ ^22^ ^]^ Despite remarkable advances, only a small subset of patients can obtain durable clinical benefits, thus substantial therapeutic challenges remain. Transcription factors (TFs), serving as gene regulatory hub, play a crucial role in orchestrating the biological behaviors of tumors.^[^ ^23^, ^24^ ^]^ Dysregulation of TFs plays an essential role in regulating tumor microenvironment (TME) and subsequently impacts tumorigenesis.^[^ ^25^, ^26^, ^27^ ^]^ Currently, the impact of m^6^A modification and m^6^A regulators on immune evasion and angiogenesis through the regulation of TFs remain unclear. Exploring the m^6^A modification on TFs may disclose new molecular mechanisms of HCC pathology and inspire promising therapeutic strategies for HCC treatment.

In the present study, we demonstrated that the expression of *YTHDF2* was aberrantly enhanced by trimethylated histone H3 lysine 4 (H3K4me3) and H3 lysine 27 acetylation (H3K27ac) modification of its promoter region, and increased *YTHDF2* expression indicated a poor prognosis for HCC patients. YTHDF2 recruited eukaryotic translation initiation factor 3 subunit B (eIF3b) to facilitate m^6^A‐modified ETS variant transcription factor 5 (ETV5) mRNA translation, thereby enhancing PD‐L1‐mediated immune evasion and VEGFA‐mediated angiogenesis. Targeting YTHDF2 by utilizing liposomes with an affinity for HCC successfully inhibited HCC growth and metastasis in an orthotopic HCC animal model.

## 2 Results

### 2.1 H3K4me3 and H3K27ac Modifications Enhance *YTHDF2* Expression and Elevated YTHDF2 Level Predicts Poor Prognosis in HCC

**Figure 1:**
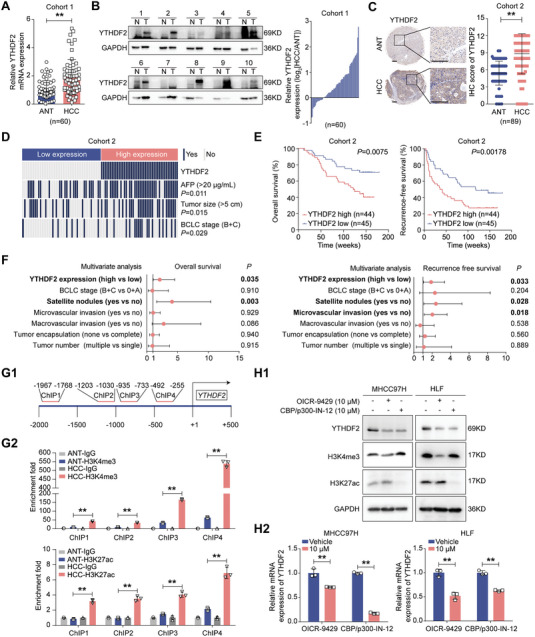
High expression of YTHDF2 is associated with HCC progression. A) The mRNA expression of YTHDF2 was analyzed by qRT‐PCR in 60 pairs of HCC samples. Data were normalized to the GAPDH and are shown as fold change relative to YTHDF2 expression of NO.1 sample. B) Representative western blotting bands of YTHDF2 in paired HCC specimens. Relative YTHDF2 protein level in HCC tissues (T) by comparing to their counterpart normal liver tissues (N) after normalizing to GAPDH expression. C) Representative IHC images and quantification analysis of YTHDF2 staining in 89 pairs of HCC; Scale bar, 200 µm in left and right images. D) Chi‐square analysis of the relevance of YTHDF2 expression with alpha‐fetoprotein level, tumor size and Barcelona Clinic Liver Cancer stage in HCC patients. E) HCC patients were divided into two groups (YTHDF2 high or low) according to their median IHC score of YTHDF2 in (C). Kaplan‐Meier analyses for overall survival and recurrence‐free survival for HCC patients in these two groups. F) Forest plot of the multivariate Cox proportional hazards model for overall survival and recurrence free survival. G) *YTHDF2* promoter structure showing ChIP‐qRT‐PCR amplified regions (G1). Quantification of H3K4me3 and H3K27ac level in *YTHDF2* promoter region in HCC and ANT tissues by ChIP‐qRT‐PCR (G2). Data were normalized to each IgG group and are shown as fold change relative to ChIP1‐IgG sample. H) MHCC97H and HLF cells were treated with OICR‐9429 (10 µM) or CBP/p300‐IN‐12 (10 µM) for 24 h. The YTHDF2 mRNA and protein levels were detected by western blotting (H1) and qRT‐PCR (H1) analyses. GAPDH as loading control for western blotting in (H1). Data were normalized to GAPDH and are shown as fold change relative to HCC cells treated with vehicle in (H1). Three independent experiments were performed in (H). Data are shown as the mean ± SD in (A, C, G2 and H2). *, p < 0.05; **, p < 0.01. Abbreviations: ANT, adjacent nontumorous tissue; HCC, hepatocellular carcinoma; AFP, Alpha‐fetoprotein; BCLC: Barcelona Clinic Liver Cancer; ChIP: chromatin immunoprecipitation.

Given the discrepancy of the reported expression pattern of YTHDF2 in HCC,^[^ ^10^, ^12^, ^15^, ^28^ ^]^ we first aimed to decipher its expression landscape and clinical values in an integrated approach. By analyzing the published datasets, we demonstrated that the mRNA and protein levels of YTHDF2 were upregulated in human HCC samples compared with normal liver samples (Figure S1A,B, Supporting Information), and high expression of YTHDF2 in HCC was associated with shorter overall survival (OS) and recurrence‐free survival (RFS) (Figure S1C,D, Supporting Information). Besides, quantitative real‐time PCR (qRT‐PCR) (for cohort 1), western blotting (for cohort 1) and immunohistochemistry (IHC) analyses (for cohort 2) in paired tumor and adjacent nontumorous tissues (ANT) confirmed the expression pattern of YTHDF2 in HCC (**Figure** **1**A–C; Figure S1E,F and Table S1, Supporting Information). Notably, YTHDF2 levels positively correlated with α‐fetal protein levels, tumor size and advanced Barcelona Clinic Liver Cancer stages (Figure 1D and Table S2, Supporting Information). In addition, patients with higher YTHDF2 expression had reduced OS and RFS than patients with lower YTHDF2 levels (Figure 1E). Cox's multivariate regression analysis indicated that higher YTHDF2 expression was an independent risk factor for shorter OS and RFS (Figure 1F and Tables S3 and S4, Supporting Information).

*YTHDF2* copy number losses were found much more common than copy number gains in TCGA datasets, and there was no significant correlation between the mRNA level and the copy number of *YTHDF2* (Figure S2A,B, Supporting Information). We thus speculated that epigenetic regulation of *YTHDF2* may be responsible for its high expression in HCC. By exploring the potential epigenetic modifications in the promoter region of *YTHDF2*, the enrichment of H3K4me3 and H3K27ac modifications were found markedly higher in liver tumor cells than in normal liver tissues in the Encyclopedia of DNA Elements database (Figure S2C, Supporting Information). ChIP‐qRT‐PCR verified that H3K4me3 and H3K27ac modifications were more enriched around the *YTHDF2* transcription start site in HCC tissues than in ANTs (Figure 1G). And both the RNA and protein levels of YTHDF2 in HCC cells were significantly downregulated after treatment with an H3K4me3 inhibitor (OICR‐9429) and an H3K27ac inhibitor (CBP/p300‐IN‐12) (Figure 1H).^[^ ^29^, ^30^ ^]^ Taken together, these results demonstrated that the enrichment of H3K4me3 and H3K27ac in the promoter region might account for the upregulation of YTHDF2 in HCC, and higher expression of YTHDF2 predicted poorer prognosis.

### 2.2 YTHDF2 Triggers Immune Evasion and Angiogenesis to Promote HCC Progression

**Figure 2:**
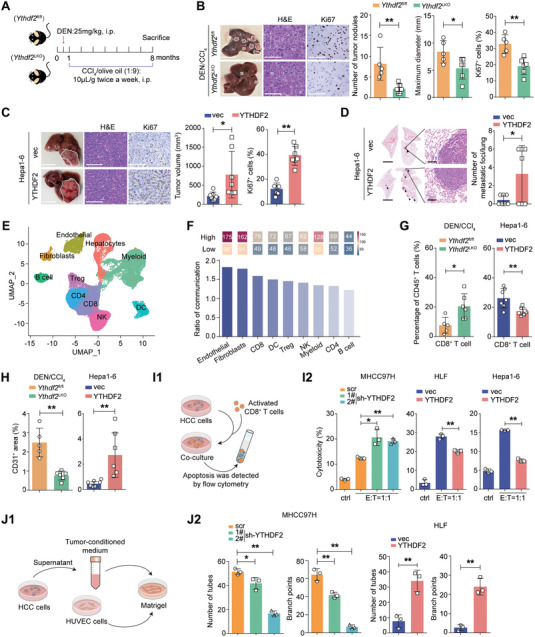
YTHDF2 provokes immune evasion and angiogenesis of HCC. A) Schematic diagram of DEN/CCl_4_ induced HCC animal model. B) Representative macroscopic, H&E and IHC staining (Ki67) images of the DEN/CCl_4_‐induced liver tumors in mice with liver‐specific knockout of *Ythdf2* (*Ythdf2^LKO^*) (n = 6) and in control mice (*Ythdf2^fl/fl^*) (n = 5). Scale bar, 1 cm for macroscopic images, 200 µm for H&E and Ki67 staining images. The tumor number, the largest tumor diameter and Ki67 staining intensity were quantified. C) Representative macroscopic, H&E and IHC staining (Ki67) images of liver tumor in mice injected with Hepa1‐6 cells orthotopically after overexpressing YTHDF2 (n = 7). Scale bar, 1 cm for macroscopic images, 200 µm for H&E and Ki67 staining images. The tumor size and Ki67 staining intensity were calculated. D) Representative H&E staining for lung metastases of mice in (C). The number of lung metastatic foci were calculated. Scale bar, 1 cm for macroscopic images, 50 µm for magnified images. E) UMAP plots for the different clusters’ identification of single cells in human HCC tumor. F) The heatmap show the communication score between YTHDF2 high/low expression HCC cell and distinct cell subpopulations (upper panel). The ratio (YTHDF2 high/low expression cell communication) were calculated (down panel). (G and H) DEN/CCl_4_‐induced (*Ythdf2* ^fl/fl^, n = 5; *Ythdf2* ^LKO^, n = 6) and Hepa1‐6 xenografted (Hepa1‐6‐vec, n = 7; Hepa1‐6‐YTHDF2, n = 7) liver tumors were subjected to flowcytometry and IHC analyses. Qualification of the ratio of tumor‐infiltrating CD8^+^ T cells among the total CD45^+^ population G). The CD31^+^ area in IHC slides was quantified H). I) Schematic diagram for CD8^+^ T cell‐mediated cytotoxicity assay (I1). The indicated HCC cells (targeted cells) were co‐cultured with CD8^+^ T cells (effector cells). The cytotoxicity of CD8^+^ T cells were determined by the apoptotic ratio of HCC cells (I2). J) Tube formation assay was performed by culturing HUVEC cells on Matrigel with the indicated tumor‐conditioned medium (J1). The number of tuber and branch points were quantified (J2). Three independent experiments were performed in I and J). Data are shown as the mean ± SD in (B‐D and G‐J). *, p < 0.05; **, p < 0.01. Abbreviations: DEN, N‐Nitrosodiethylamine; CCl_4_: carbon tetrachloride; fl/fl, flox/flox; H&E: hematoxylin and eosin; LKO, liver specific knockout; i.p., intraperitoneal injection; vec, vector; sh, small hairpin RNA; OE, overexpression; T, targeted cells; E, effector cells.

To figure out the functions of YTHDF2 in HCC formation and progression, we generated hepatocyte‐specific *Ythdf2*‐knockout mice (*Ythdf2* ^LKO^) by crossing albumin‐Cre mice with *Ythdf2* ^fl/fl^ mice (Figure S3A,B, Supporting Information) and administered diethylnitrosamine (DEN)/carbon tetrachloride (CCl_4_) to induce HCC formation (**Figure** **2**A). Compared with *Ythdf2* ^fl/fl^ littermates, *Ythdf2* ^LKO^ mice exhibited less tumor burden, as evidenced by reduced tumor number, decreased maximum tumor diameter and weaker proliferation activity (Figure 2B). Next, a xenograft mice model was established by inoculating mice‐derived HCC cells, namely, Hepa1‐6 cells, into the liver. The volume and proliferation activity of tumor nodules, as well as lung metastatic foci, were significantly higher in mice that were injected with Hepa1‐6 cells overexpressing YTHDF2 (Figure 2C,D; Figure S3C, Supporting Information).

To explore how YTHDF2 modulate the interaction between HCC cells and tumor microenvironment, we analysis the single cell RNA‐sequencing data of GSE202642 (Figure 2E). Interestingly, HCC cells with high expression of YTHDF2 intensively interacted with endothelial, fibroblastic cells, as well as CD8^+^ T cells (Figure 2F). Given the critical role of CD8^+^ T cells and vascular endothelial cells in HCC formation and progression,^[^ ^17^, ^18^ ^]^ we focused on potential effects of YTHDF2 on these two cell clusters. Flow cytometry analysis showed that YTHDF2 inhibited the accumulation of CD8^+^ T cells in both DEN/CCl_4_‐induced and Hepa1‐6 xenografted liver tumors (Figure 2G; Figure S4A, Supporting Information). And, endothelial cell adhesion molecule‐1 (CD31) staining, a marker for vascular endothelial cell, was decreased in the tumors of *Ythdf2* ^LKO^ mice but was elevated in the xenografted tumors with YTHDF2 overexpression (Figure 2H; Figure S4B, Supporting Information). At the same time, in vitro assays demonstrated that HCC cells with higher YTHDF2 level displayed stronger resistance to CD8^+^ T cells mediated cytotoxicity, with increasing abilities to inducing angiogenesis (Figure 2I,J; Figure S4C,D, Supporting Information).

### 2.3 PD‐L1 and VEGFA Mediate the Tumor Promoting Role of YTHDF2

**Figure 3:**
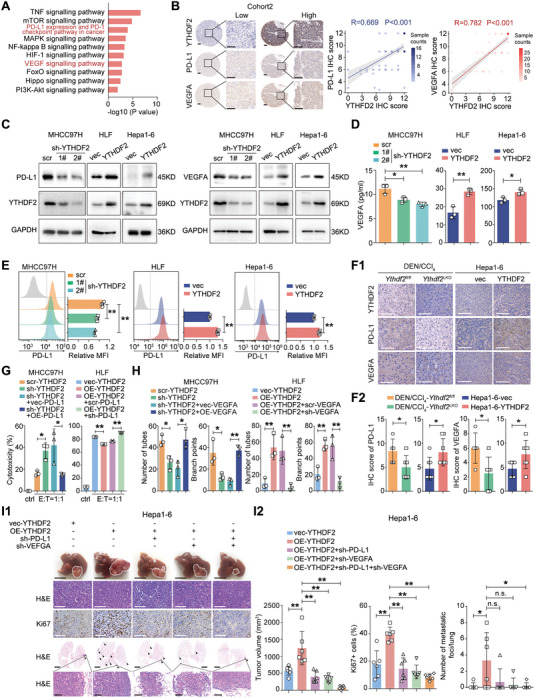
YTHDF2 promotes HCC progression by upregulating PD‐L1 and VEGFA expression. A) The genes (Pearson≥0.3 or ≤−0.3, P<0.05) correlated with YTHDF2 expression in TCGA‐LIHC dataset were subjected to KEGG analysis. B) Representative images of IHC staining of YTHDF2, PD‐L1 and VEGFA in HCC samples. Scale bar, 200 µm for overview and magnified images. Spearman correlation analysis between YTHDF2 and PD‐L1/VEGFA in 89 pairs of HCC samples. C) Western blotting analysis for PD‐L1 and VEGFA expression in HCC cells with YTHDF2 knocked down and overexpression. GAPDH as loading control. D) ELISA assay for VEGFA level in the supernatants of the indicated HCC cells. E) Flow cytometry analysis for cell surface located PD‐L1 expression in the indicated HCC cells. F) Representative images and quantification of IHC staining for PD‐L1 and VEGFA in the DEN/CCl_4_‐induced (*Ythdf2* ^fl/fl^, n = 5; *Ythdf2* ^LKO^, n = 6) and Hepa1‐6 xenografted (Hepa1‐6‐vec, n = 7; Hepa1‐6‐YTHDF2, n = 7) liver tumors. Scale bar, 200 µm. G) PD‐L1 was overexpressed or knocked down in YTHDF2 knocked down/overexpressed HCC cells. The indicated HCC cells (targeted cells) were co‐cultured with CD8^+^ T cells (effector cells) at the indicated ratio. The cytotoxicity of CD8^+^ T cells were determined by the apoptotic ratio of HCC cells. H) Tube formation assay was performed, and the number of tubes and branch points were quantified. I) PD‐L1 and VEGFA were knocked down in HCC cells with YTHDF2 overexpression. The indicated HCC cells were implanted in mice liver. Representative images of liver tumor and lung metastatic foci (I1). Tumor volume, Ki67 staining intensity and lung metastases foci were quantified (I2). HCC cells were treated with IFN‐γ (20 ng mL^−1^) for 24 h to detect the expression of PD‐L1 in (C and E). Data are shown as the mean ± SD. Three independent experiments were performed for (D, E, G and H). *, p < 0.05; **, p < 0.01. Abbreviations: MFI; mean fluorescent intensity; scr, scramble; PD‐L1, programmed death ligand‐1; VEGFA, vascular endothelial growth factor A.

To explore by which YTHDF2 modulate immune evasion and angiogenesis in HCC, we analyzed the genes whose expression correlated with YTHDF2 expression in the TCGA database (Pearson≥0.3, or ≤−0.3, P<0.05). The Kyoto Encyclopedia of Genes and Genomes (KEGG) analysis showed that those genes were enriched in many cancer‐related signaling pathways, including “PD‐L1 expression and PD‐1 checkpoint pathway in cancer” and “VEGFA‐signaling pathway” (**Figure** **3** **A**). Moreover, among the genes enriched in these two pathways, PD‐L1 and VEGFA, which are critical for mediating immune evasion and angiogenesis in HCC,^[^ ^18^, ^31^ ^]^ were found positively correlated with YTHDF2 expression (Figure 3B; Figure S5A–C, Supporting Information). Western blotting analysis showed that endogenous expression of YTHDF2 was distinct in HCC cells (Figure S5D, Supporting Information). Thus, we knocked down YTHDF2 in MHCC97H cells (MHCC97H/sh‐YTHDF2) and overexpressed YTHDF2 in HLF and Hepa1‐6 cells (HLF/OE‐YTHDF2 and Hepa1‐6/OE‐YTHDF2). The qRT‐PCR and western blotting analyses indicated that knocking down YTHDF2 in MHCC97H cells downregulated PD‐L1 and VEGFA expression, and opposite results were observed in HLF/OE‐YTHDF2 and Hepa1‐6/OE‐YTHDF2 cells (Figure 3C; Figure S5E, Supporting Information). In addition, flow cytometry and enzyme‐linked immunosorbent assay (ELISA) showed that cell surface‐localized PD‐L1 (sPD‐L1) and the secreted form of VEGFA (sVEGFA) were significantly lower in HCC cells with relatively lower YTHDF2 expression (Figure 3D,E). Similar results were observed in IHC staining for PD‐L1 and VEGFA in DEN/CCl_4_‐induced and orthotopic HCC tumors (Figure 3F).

Additionally, the enhanced killing ability of CD8^+^ T cells induced by YTHDF2 knockdown in HCC cells was reversed by overexpressing PD‐L1, and opposite trends were observed in HLF/OE‐YTHDF2 and Hepa1‐6/OE‐YTHDF2 cells (Figure 3G; Figure S5F,G, Supporting Information). In addition, we found that the impaired tube formation ability in human umbilical vein endothelial cells (HUVECs) that were cultured with MHCC97H/sh‐YTHDF2 cell supernatants was recovered by VEGFA overexpression, and opposite results were observed in HLF/OE‐YTHDF2 cells (Figure 3H; Figure S5H,I, Supporting Information). Moreover, the increased tumor size and lung metastatic foci induced by YTHDF2 overexpression were impaired after inhibiting PD‐L1 or VEGFA expression in Hepa1‐6 cells, and these effects were most obvious when both PD‐L1 and VEGFA were downregulated (Figure 3I; Figure S5J, Supporting Information). These results indicated that YTHDF2 promotes immune evasion and angiogenesis via PD‐L1 and VEGFA in HCC.

### 2.4 ETV5 is the Direct Downstream Target of YTHDF2

**Figure 4:**
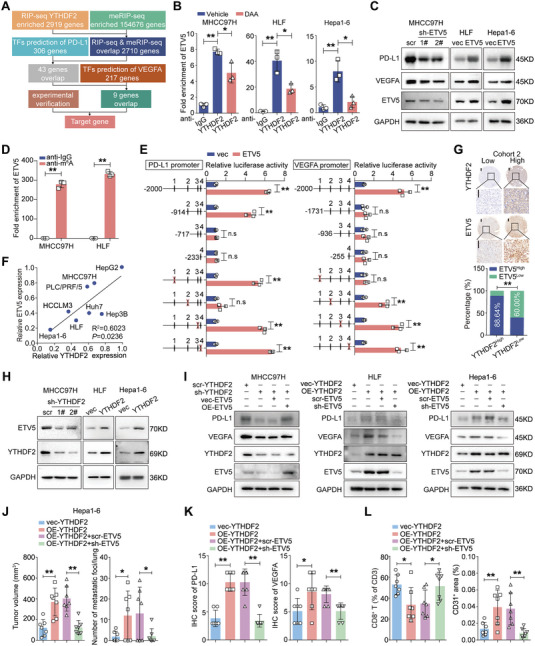
ETV5 mediates the regulation of YTHDF2 on PD‐L1/VEGF1 by enhancing their transcription. A) Schematic workflow for screening direct targets of YTHDF2. B) The qRT‐PCR analysis for the enrichment of ETV5 mRNA in anti‐YTHDF2 RNA precipitates in HCC cells with or without DAA treatment. Data are shown as fold change relative to anti‐IgG. C) The western blotting analyses for the protein level of PD‐L1 and VEGFA in HCC cells with YTHDF2 stably overexpressed or knocked down. Data were normalized to GAPDH and are shown as fold changes relative to scramble (scr) or vector (vec) groups. D) The qRT‐PCR analysis for the ETV5 mRNA level in anti‐m^6^A RNA precipitates. Data are shown as fold change to anti‐IgG group. E) Schematic diagram of pGL4.17 based PD‐L1/VEGFA promoter reporter constructs containing truncates and mutates for the four putative ETV5‐binding sites located in the promoter regions (left). The transcriptional activity of the luciferase reporter constructs with wild type and mutant ETV5‐binding sites were determined by dual‐luciferase assay in ETV5 overexpressing HLF cells (right). Data are shown as fold change relative to their respective HLF/vec groups. F) Pearson's correlation analysis of the protein expression level of YTHDF2 and ETV5 in HCC cell lines. G) Representative IHC images for ETV5 and YTHDF2 in HCC samples. Scale bar, 200 µm in overview and magnified images. Chi‐square analysis demonstrated the positive correlation between the expression level of ETV5 and YTHDF2 in HCC tissues. H) Western blotting analysis for ETV5 expression in HCC cells with YTHDF2 overexpressed or knocked down. I) The expression of PD‐L1 and VEGFA were detected by western blotting in YTHDF2 knocked down/overexpressed HCC cells with ETV5 overexpressed/knocked down. HCC cells were treated with IFN‐γ (20 ng mL^−1^) for 24 h to detect the expression and transcriptional activity of PD‐L1. J‐I) Hepa1‐6 cells with YTHDF2 stably overexpressed (OE‐YTHDF2) with or without ETV5 knocked down (sh‐ETV5) were inoculated in the mice liver (n = 7 for each group). The tumor volume and lung metastatic foci were calculated J). Quantification of IHC staining intensity for each protein K). Flow cytometry analysis for tumor‐infiltrating CD8^+^ T cells in the indicated orthotopic liver tumors and quantification of CD31^+^ area in orthotopic liver tumors I). GAPDH as loading control in C, H and I). Data are shown as the mean ± SD in B, E and J–L). Three independent experiments were performed for B–D, E, H and I). *, p < 0.05; **, p < 0.01. n.s., no significance. Abbreviations: RIP‐seq, RNA immunoprecipitation‐sequencing; meRIP, m^6^A specific methylated RNA immunoprecipitation; TFs, transcription factors.

RNA immunoprecipitation (RIP) analysis showed that anti‐YTHDF2 antibodies failed to enrich PD‐L1/VEGFA mRNA from MHCC97H cells (Figure S6A, Supporting Information). In addition, knocking down YTHDF2 exerted no effect on PD‐L1/VEGFA mRNA degradation, which was considered the main effect of YTHDF2 on the fate of mRNAs (Figure S6B, Supporting Information).^[^ ^7^ ^]^ These results suggested that PD‐L1 and VEGFA mRNA transcripts may not be the direct targets of YTHDF2. To explore the downstream targets of YTHDF2, we conducted m^6^A‐specific methylated RNA immunoprecipitation (meRIP)‐seq and RIP‐seq in MHCC97H cells. Global profiling of m^6^A‐modified mRNA was performed by meRIP‐seq (Table S5, Supporting Information). Consistent with previous reports, a “‘GGAC”’ sequence motif was enriched in the immunopurified RNA (Figure S6C, Supporting Information). GO analysis for RIP‐seq (Table S6, Supporting Information) suggested that the RNAs enriched by YTHDF2 were generally involved in DNA transcription (Figure S6D, Supporting Information), which was consistent with previous reports.^[^ ^32^ ^]^ Thus, we hypothesized that YTHDF2 regulates PD‐L1/VEGFA expression through m^6^A‐modified transcription factors. Taking the intersection of RIP‐seq results, meRIP‐seq results and the transcription factors of PD‐L1/VEGFA that were predicted by JASPER, 9 genes were included (**Figure** **4**A). The interactions between the YTHDF2 protein and ETV5 mRNA, together with the other 6 mRNAs, were confirmed by RIP‐qRT‐PCR. In addition, inhibiting the m^6^A modification inhibited the binding of YTHDF2 and these mRNAs (Figure 4B; Figure S6E, Supporting Information). Among the 7 genes, only ETV5 regulated both PD‐L1 and VEGFA expression, and the m^6^A modification in ETV5 mRNA was validated by meRIP analysis (Figure 4C,D; Figure S6F,G and Table S7, Supporting Information). Four putative ETV5 binding sites (BSs) were predicted in the PD‐L1/VEGFA promoter region. Dual luciferase reporter assays by mutating potential BSs demonstrated that BS2/BS1 was indispensable for PD‐L1/VEGFA reporter activity (Figure 4E). Moreover, chromatin immunoprecipitation (ChIP)‐qRT‐PCR assays showed that ETV5 bound to the BS2 of the PD‐L1 promoter and BS1 of the VEGFA promoter (Figure S6H, Supporting Information).

Thus, we speculated that ETV5 was the direct target of YTHDF2 in HCC cells. Western blotting analysis showed that the expression of ETV5 was positively correlated with YTHDF2 levels in HCC cell lines (Figure 4F; Figures S7A and S5C, Supporting Information). Chi‐square analysis of IHC staining showed that the expression levels of YTHDF2 and ETV5 were positively correlated in HCC tissues (Figure 4G; Figure S7B, Supporting Information). Additionally, patients with HCC with higher YTHDF2 and higher ETV5 expression had significantly worse survival (Figure S7C, Supporting Information). Overexpression of YTHDF2 increased the protein level of ETV5, and vice versa in HCC cell lines (Figure 4H), despite the negative regulation of ETV5 mRNA expression by YTHDF2 (Figure S7D,E, Supporting Information).

To further validate the role of ETV5 in the YTHDF2‐mediated promotion of HCC progression, rescue assays were performed by recovering ETV5 expression in YTHDF2 knocked down and overexpressed HCC cells. The YTHDF2 knockdown‐induced PD‐L1 and VEGFA downregulation was reversed by ETV5 overexpression in MHCC97H cells. Similar results were obtained in ETV5‐knockdown HLF/OE‐YTHDF2 and Hepa1‐6/OE‐YTHDF2 cells (Figure 4I). In vitro T‐cell cytotoxicity assays showed that Hepa1‐6/OE‐YTHDF2 cells were less sensitive to CD8^+^ T‐cell killing, whereas knocking down ETV5 attenuated this effect (Figure S7F, Supporting Information). In addition, ETV5 knockdown reversed the enhanced tube formation capacity of HUVECs that were cultured with HLF/OE‐YTHDF2 cells (Figure S7G, Supporting Information). Hepa1‐6/OE‐YTHDF2 cells with ETV5 knocked‐down were then injected into mice livers. The increased tumor size and lung metastatic foci induced by YTHDF2 overexpression were reversed after ETV5 was downregulated (Figure 4J; Figure S7H, Supporting Information). IHC analysis showed that the increased expression of PD‐L1 and VEGFA in YTHDF2‐overexpressing Hepa1‐6 tumors was downregulated after knocking down ETV5 (Figure 4K; Figure S7H, Supporting Information). Moreover, the decrease in CD8^+^ T‐cell infiltration and enhanced angiogenesis in Hepa1‐6/OE‐YTHDF2 tumors were reversed by knocking down ETV5 expression (Figure 4L; Figure S7I, Supporting Information).

### 2.5 YTHDF2 Facilitates the Translational Process of ETV5 mRNA in an m^6^A Dependent Manner

**Figure 5:**
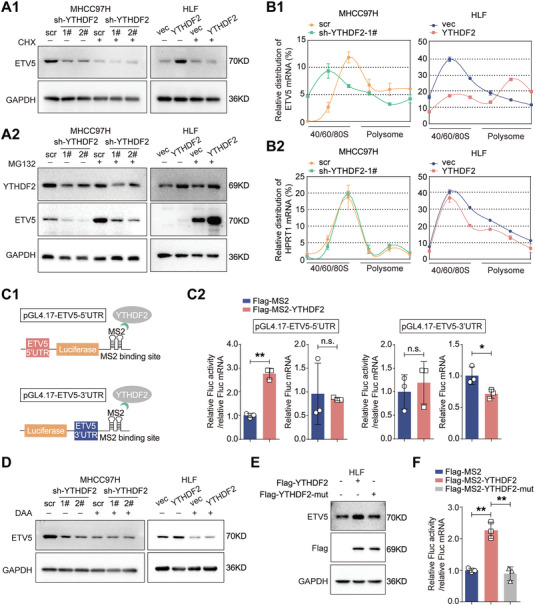
YTHDF2 facilitates the translation of ETV5 mRNA by recognizing the m^6^A‐modification. A) The level of ETV5 was detected by western blotting in the indicated HCC cells with or without CHX (A1) or MG132 (A2) treatment. B) Relative levels of ETV5 and HPRT1 mRNAs in different ribosome fraction were evaluated by qRT‐PCR analysis. Data are plotted as a percentage of the total RNA. C) Schematic representation of pGL4.17‐ETV5‐5′UTR and pGL4.17‐ETV5‐3′UTR of tethering assay (C1). The translation efficiency and the relative Firefly luciferase (FLuc) mRNA expression of pGL4.17‐ETV5‐5′UTR and pGL4.17‐ETV5‐3′UTR in indicated HLF cells (C2). D) Western blotting analysis for ETV5 expression in the indicated HCC cells with or without DAA treatment. E) HCC cells were transfected with plasmids transiently expressing Flag‐YTHDF2 or Flag‐YTHDF2‐mut (K416A, R527A, W432A, W486A and W491A) for 48 h. Western blotting analysis for ETV5 expression in the indicated HCC cells. F) The translation efficiency of pGL4.17‐ETV5‐5′UTR in indicated HLF cells. FLuc activity was measured and normalized to the Renilla luciferase (RLuc) activity. Relative FLuc activity was normalized to the relative FLucMS2bs mRNAs (C and F). GAPDH as loading control in (A, D and E). Data are shown as the mean ± SD for three independent experiments in (B, C and F). *, p < 0.05; **, p < 0.01. Abbreviations: CHX: cycloheximide; DAA: 3‐deazaadenosine. 5′UTR, 5′ untranslated region; 3′UTR, 3′ untranslated region.

Divergences in the regulation of protein and RNA levels (Figure 4H; Figure S7C, Supporting Information) suggested that YTHDF2 increased the protein expression of ETV5 independently of its canonical function. Treatment of cells with cycloheximide (CHX) but not MG132 significantly diminished the effects of YTHDF2 on ETV5 protein levels (**Figure** **5**A). Polysome profiling coupled with qRT‐PCR verified that the relative distribution of ETV5 mRNA, but not hypoxanthine phosphoribosyltransferase 1 (HPRT1, a nonm^6^A‐modified mRNA), was shifted from polysome (translation active) toward subpolysome (translation inactive) fractions in YTHDF2‐knockdown HCC cells, and vice versa (Figure 5B; Figure S8A, Supporting Information). These results indicated that YTHDF2 may increase the overall protein expression of ETV5 by facilitating the translation of ETV5 mRNA. MeRIP‐seq and previous research showed that the m^6^A peaks were predominantly localized near stop codons in the 3′ untranslational region (UTR), with a subset of m^6^A peaks located in the 5′UTR (Figures S8B, Supporting Information).^[^ ^6^, ^33^ ^]^ To investigate whether m^6^A‐methylated 5′UTR and/or 3′UTR were involved in the YTHDF2‐regulated translation of ETV5 mRNA, we constructed luciferase reporter plasmids containing two MS2‐binding sites to perform a tethering assay (Figure 5C1, Supporting Information). The translation efficiency of pGL4.17‐ETV5‐5′UTR, rather than pGL4.17‐ETV5‐3′UTR, in HLF/OE‐YTHDF2 cells was significantly increased (Figure 5C2; Figure S8C, Supporting Information).

Given that YTHDF2 specifically binds to m^6^A modifications in transcripts,^[^ ^34^ ^]^ we proposed that YTHDF2 regulated ETV5 expression in an m^6^A‐dependent manner. Indeed, inhibiting m^6^A modification by 3‐deazaadenosine (DAA) treatment attenuated the regulation of ETV5 expression by YTHDF2 in the indicated HCC cells (Figure 5D). To abolish the capacity of YTHDF2 to recognize and bind to m^6^A modifications, we introduced five key point mutations (K416A, R527A, W432A, W486A and W491A) into YTHDF2 (YTHDF2‐mut).^[^ ^35^ ^]^ Compared with wild‐type YTHDF2 fragments, YTHDF2‐mut failed to upregulate ETV5 protein levels (Figure 5E). In addition, YTHDF2‐mut failed to enhance the translational activity of pGL4.17‐ETV5‐5′UTR (Figure 5F). Thus, these results demonstrate that YTHDF2 regulates the translational efficiency of ETV5 mRNA in an m^6^A‐dependent manner and results in increased overall ETV5 protein expression.

### 2.6 EIF3b is Indispensable for the Enhanced Translation of ETV5 mRNA Induced by YTHDF2

**Figure 6:**
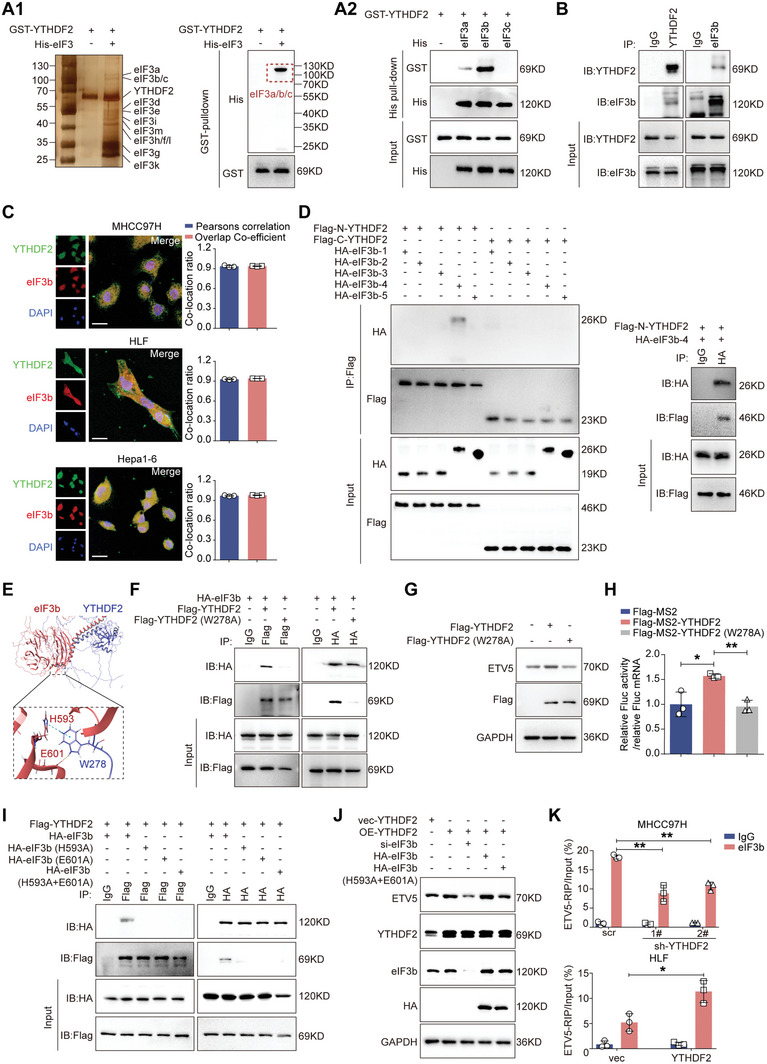
EIF3b is indispensable for YTHDF2‐enhanced translation of ETV5 mRNA. A) Sliver‐stained SDS‐PAGE gel of purified His‐labelled eIF3 subunit proteins (His‐eIF3) and GST‐labelled YTHDF2 (GST‐YTHDF2) (left panel). The direct interaction between GST‐YTHDF2 and the His‐eIF3 was evaluated by GST‐pulldown assay (A1). His‐pulldown assay for detecting the binding between GST‐YTHDF2 with His‐eIF3a/b/c (A2). B) The binding between YTHDF2 and eIF3b in MHCC97H cells was detected by endogenous co‐immunoprecipitation assay. C) Representative confocal images of YTHDF2 and eIF3b. Green, YTHDF2; Red, eIF3b; Blue, DAPI. Scale bar, 10 µm. Immunofluorescence revealing co‐localization of YTHDF2 and eIF3b by detecting Pearsons's correlation and overlap co‐efficient. D) HEK‐293T cells were transfected with plasmids expressing the indicated truncations of YTHDF2 and eIF3b for 48 h. Identification of the interaction between truncated eIF3b and truncated YTHDF2 by the co‐IP assays. E) Molecular docking of 3D structures shows the interaction of W278 site of YTHDF2 (blue) with H593 and E601 sites of eIF3b (red). F) HEK‐293T cells were co‐transfected with plasmids expressing HA‐labelled eIF3b and Flag‐labelled YTHDF2 or Flag‐labelled YTHDF2 (W278A) for 48 h. Co‐IP assays show the interaction between W278A mutated YTHDF2 and eIF3b. G) The level of ETV5 was detected by western blotting in the indicated HCC cells transfected with YTHDF2 or W278A mutated YTHDF2. H) The translation efficiency of pGL4.17‐ETV5‐5′UTR in indicated HLF cells. FLuc activity was measured and normalized to the Renilla luciferase (RLuc) activity. Relative FLuc activity was normalized to the relative FLucMS2bs mRNAs. I) HEK‐293T cells were co‐transfected with plasmids expressing Flag‐labelled YTHDF2 and HA‐labelled eIF3b or its mutants (H593A, E601A and H593A+H601A) for 48 h. Co‐IP assays show the interaction between mutated eIF3b and YTHDF2. J) SiRNAs targeting eIF3b were transfected into YTHDF2 overexpressed HCC cell, with co‐transfection of plasmids expressing HA‐eIF3b or HA‐eIF3b (H593A+H601A). Western blotting analysis for ETV5 expression. K) The qRT‐PCR analysis for the enrichment of ETV5 mRNA in anti‐eIF3b precipitates after knocking down/overexpressing YTHDF2 in HCC cells. Data are shown as fold changes relative to their respective anti‐IgG groups. GAPDH as loading control in (G and J). Data are shown as the mean ± SD in (G and J). Three independent experiments were performed. *, p < 0.05; **, p < 0.01. Abbreviations: M, molecular weight markers; IP, immunoprecipitation; IB, immunoblotting.

As the largest initiation factor, eIF3 plays a pivotal role in acting as a scaffold for the recruitment of mRNA, 40S subunits and other eIFs, and it is indispensable for stimulating nearly all steps of translation initiation.^[^ ^36^ ^]^ GST pull‐down analysis suggested that YTHDF2 may directly and specifically bind to eIF3a/b/c (**Figure** **6A1**). Next, a strong interaction between YTHDF2 and eIF3b was verified by His‐pulldown (Figure 6A2). The binding of endogenous YTHDF2 and eIF3b in HCC cells was assessed by coimmunoprecipitation (co‐IP) (Figure 6B). In situ fluorescence staining revealed the colocalization of YTHDF2 and eIF3b in HCC cells (Figure 6C). In addition, exogenous co‐IP analysis indicated that their interaction was RNA‐independent (Figure S9A, Supporting Information).

To map the binding domain of YTHDF2 that is responsible for the interaction with eIF3b, we generated Flag‐tagged YTHDF2 truncated mutants and HA‐tagged eIF3b truncated mutants (Figure S9B, Supporting Information). Co‐IP demonstrated that the N‐terminal of YTHDF2 specifically interacted with the eIF3b 426–642 amino acid sequence (eIF3b‐4) (Figure 6D). To predict the binding sites that mediate this interaction, we performed molecular docking via ZDOCK calculations, which showed that the W278 site of YTHDF2 may be critical for binding to the H593 and E601 sites of eIF3b (Figure 6E). To test this hypothesis, all of these sites were individually mutated to alanine, and these mutants were transiently transfected into HEK‐293T cells to examine the interaction between YTHDF2 and eIF3b. The results showed that the W278A mutation of YTHDF2 abolished the interaction between YTHDF2 and eIF3b (Figure 6F). In addition, the W278A mutation of YTHDF2 markedly reduced its ability to promote translation of ETV5 and failed to upregulate ETV5 expression (Figure 6G,H; Figure S9C, Supporting Information). Additionally, both eIF3b (H593A) and eIF3b (E601A) significantly abrogated the binding of YTHDF2 to eIF3b (Figure 6I). Moreover, the YTHDF2 overexpression‐induced increase in ETV5 expression was reversed after eIF3b was downregulated, but overexpressing wild‐type eIF3b, rather than eIF3b (H593A+E601A), recovered the regulatory effect of YTHDF2 on ETV5 (Figure 6J). The RIP assay showed that the abundance of ETV5 mRNA in the anti‐eIF3b precipitate was decreased in the YTHDF2‐knockdown MHCC97H cells, but it was increased in the YTHDF2‐overexpressing HLF cells (Figure 6K). These results indicated that YTHDF2 facilitates ETV5 mRNA translation by promoting the interaction between eIF3b and ETV5 mRNA.

### 2.7 Targeting YTHDF2 Inhibits HCC Progression In Vivo

**Figure 7:**
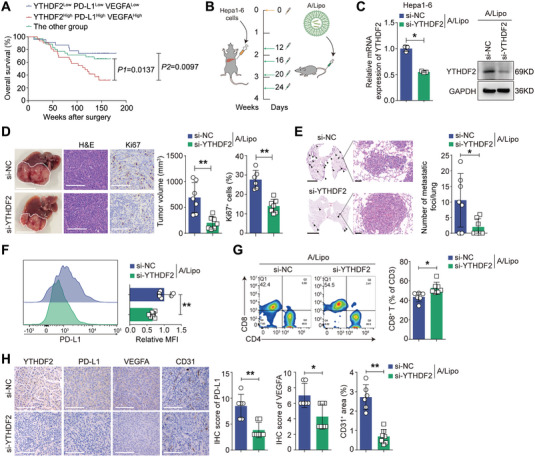
Targeting YTHDF2 inhibits HCC progression in vivo. A) The 89 HCC patients in cohort 2 were stratified into three groups according to the IHC score of YTHDF2, PD‐L1 and VEGFA. Kaplan‐Meier analysis for the overall survival in the three groups. B) Schematic diagram of orthotopic HCC animal model treated with A/Lipo/si‐YTHDF2 complex. C) The knocking down efficiency of A/Lipo/si‐YTHDF2 complexes was evaluated by qRT‐PCR and western blotting analyses. Data were normalized to GAPDH and are shown as fold change relative to A/Lipo/si‐NC group. GAPDH as loading control for western blotting. D) Representative images of liver tumor (Scale bar, 1 cm), H&E staining (Scale bar, 200 µm) and IHC staining for Ki67 (Scale bar, 200 µm). Orthotopic tumor volume and Ki67 staining intensity were quantified. E) Representative images of H&E staining for lung metastases. Scale bar, 1 cm for macroscopic images, 50 µm for magnified images. The number of lung metastatic foci in each group were calculated. (F and G) Flowcytometry analysis for the cell‐surface PD‐L1 level F) and tumor‐infiltrating CD8^+^ T cells G) in the liver tumors treated with different A/lipo/siRNA complexes. H) Representative IHC images of YTHDF2, PD‐L1, VEGFA and CD31 in orthotopic liver tumors treated with the indicated A/Lipo/siRNA complexes. Scale bar, 200 µm. The IHC intensity for each protein were quantified. *, p < 0.05; **, p < 0.01. Data are shown as the mean ± SD in (C‐H). Abbreviations: A/Lipo: aptamer/liposomes.

According to the median IHC scores of YTHDF2, PD‐L1 and VEGFA, we classified HCC patients into three subgroups: 1) YTHDF2^low^, PD‐L1^low^ and VEGFA^low^ (n = 22); 2) YTHDF2^high^, PD‐L1^high^ and VEGFA^high^ (n = 31); and 3) “the other group” (n = 36), which included the remaining samples. Kaplan‐Meier analysis revealed that Group 2 exhibited a worse prognosis (**Figure** **7**A). Given the regulation of YTHDF2 on the expression of PD‐L1 and VEGFA, we aimed to determine whether YTHDF2 could be a potential target in the treatment of HCC. An aptamer/liposome (A/Lipo) complex with an affinity for HCC was used to deliver small interference RNA targeting YTHDF2 (A/Lipo/si‐YTHDF2) to the livers of the orthotopic HCC model (Figure 7B).^[^ ^37^ ^]^ The dispersity of A/Lipo/si‐YTHDF2 (Figure S10A, Supporting Information) and its knockdown efficiency (Figure 7C) were confirmed. To detect the specificity, we stained A/lipo complex with PKH26. The results demonstrated that A/lipo complex has a stronger affinity for HCC cells (Hepa1‐6) compared to normal liver cells (AML12) in vitro (Figure S10B, Supporting Information). In mice with orthotopic xenografts of Hepa1‐6 cells, bioluminescent images showed that DiR‐stained A/Lipo was specifically aggregated in the HCC area (Figure S10C, Supporting Information). After tail vein injection for 1 h, most fluorescence of A/Lipo/siRNAs was observed in the liver (Figure S10D, Supporting Information). Besides, A/Lipo/si‐YTHDF2 specifically inhibits the expression of YTHDF2 in tumor tissues, without affecting normal liver tissues and other organs (Figure S10E,F, Supporting Information). These results demonstrated that A/lipo/si‐YTHDF2 exhibits excellent specificity in knocking down YTHDF2 in HCC. The liver tumor burden and lung metastatic foci were lower in mice that were treated with A/Lipo/si‐YTHDF2 compared with the control group, with negligible changes in mice body weight (Figure 7D,E; Figure S10G, Supporting Information). Additionally, a significant reduction in PD‐L1 levels and increased infiltration of CD8^+^ T cells were observed in the A/Lipo/si‐YTHDF2 groups (Figure 7F,G). IHC analysis revealed lower expression of PD‐L1, VEGFA and CD31 in A/Lipo si‐YTHDF2‐treated tumors than in control tumors (Figure 7H).

## 3 Discussion

The role of YTHDF2 in HCC has been intensively investigated, and inconsistent conclusions have been drawn. Jiajie Hou et al. reported that YTHDF2 was downregulated in HCC and that low expression of YTHDF2 predicted worse prognosis. Hypoxia in the tumor mass decreased YTHDF2 expression to induce interleukin 11‐mediated inflammation‐cancer formation processes and serpin family E member 2‐mediated disruption of vascular normalization, thereby facilitating HCC formation and metastasis.^[^ ^15^ ^]^ Additionally, hypoxia‐induced YTHDF2 expression inhibited cell proliferation and growth by accelerating the degradation of epidermal growth factor receptor mRNA.^[^ ^16^ ^]^ However, Yang et al. demonstrated that HBV infection led to the enhanced O‐GlcNAcylation of YTHDF2, thereby upregulating its level by preventing its ubiquitination‐mediated degradation in HCC. Enhanced YTHDF2 expression promoted HCC proliferation by preserving the stability of the minichromosome maintenance protein 2 and minichromosome maintenance protein 5 transcripts.^[^ ^12^ ^]^ In addition, liver cancer stem cell phenotypes were reported to be enhanced by YTHDF2, and YTHDF2 promoted cancer metastasis by facilitating organic cation/carnitine transporter 4 translation.^[^ ^10^ ^]^ In this report, we investigated the clinical relevance of YTHDF2 in HCC by integrated approaches using public transcriptome and proteome sequencing data, qRT‐PCR and western blotting analyses of snap‐frozen HCC tissues, and in situ IHC staining of formalin‐fixed paraffin‐embedded HCC specimens. We revealed that the epigenetic regulation of *YTHDF2*, specifically by H3K4me3 and H3K27ac in the DNA promoter region, contributed to the elevated expression of YTHDF2 in HCC. Gain‐ and loss‐of‐function assays revealed its role as a tumor promoter by promoting immune evasion and angiogenesis by PD‐L1 and VEGFA in HCC.

Previous reports suggested that elevated mRNA levels of genes in tumor cells do not necessarily correspond to increased protein translation and pointed out the conflicts between transcription and translation processes.^[^ ^38^ ^]^ Herein, we proposed “degradation and translation conflicts” in the regulation of mRNAs by YTHDF2. In detail, we found that the mRNA level of ETV5 was negatively correlated with the level of YTHDF2 in HCC, and YTHDF2 shortened the half‐life of ETV5 mRNA by recognizing m^6^A sites in a CCR4‐NOT1‐dependent manner (data not shown). Interestingly, the protein level of ETV5 was positively correlated with the level of YTHDF2 in HCC. Furthermore, we revealed that YTHDF2 recognized the m^6^A sites in the 5′UTR and recruited eIF3b to facilitate the translation of ETV5 mRNA. The overall effects of “degradation and translation conflicts” caused by YTHDF2 resulted in the elevated protein expression of ETV5 and promoted tumor progression. The discrepancy in the regulation of the different processes of an oncogenic mRNA raised the possibility that the dual effects of YTHDF2 (as a promoter of mRNA degradation and a facilitator of translation) in cancers are exerted via its regulation of the different processes related to mRNA biology. In different cellular contexts, the poised balance between mRNA degradation and translation may be altered and the role of YTHDF2 may shift from a tumor‐promoting role to a tumor‐suppressing role. Elucidating the regions of YTHDF2 that participate in its oncogenic and tumor‐suppressive roles is needed to further understand the role of YTHDF2 in cancer processes. Additionally, given the potential regulatory crosstalk between YTHDF2 and other m^6^A readers,^[39^ ^]^ it is arbitrary to assert that the stronger effect on “translation” relative to that on “degradation” is directly caused by the interaction between YTHDF2 and ETV5 mRNA.

In eukaryotic cells, initiation efficiency is considered the rate‐limiting step of translation,^[40^ ^]^ and studies have reported that many m^6^A regulatory factors facilitate translation by interacting with translation initiation factors.^[^ ^41^ ^]^ METTL3 enhances translation only when tethered to reporter mRNA at sites close to the stop codon, and the METTL3‐eIF3h interaction is required for enhanced translation, the formation of densely packed polyribosomes and oncogenic transformation.^[^ ^42^ ^]^ METTL16 promotes the translation of thousands of mRNA transcripts in the cytosol via the recruitment of eIF3a/b and ribosomal RNAs to facilitate the formation of the 40S preinitiation complex and 80S translation‐initiation complex.^[^ ^43^ ^]^ YTHDF1 can bind to m^6^A‐modified mRNA of eIF3c, consequently enhancing translation.^[^ ^44^ ^]^ In addition, eIF2AK2 bridges YTHDF3 and eIF3a, enhancing the stability of the YTHDF3/eIF3a complex to facilitate the translation of target genes.^[^ ^45^ ^]^ Under heat shock stress conditions, YTHDF2 translocates to the nucleus to protect m^6^A motifs in the 5′UTR of stress‐induced transcripts and activates cap‐independent translation initiation.^[^ ^46^ ^]^ Here, we reported that the role of YTHDF2 in promoting the translation of m^6^A‐motified ETV5 mRNA relies on eIF3b, the main scaffolding subunit in the eIF3 complex,^[^ ^47^ ^]^ and the W278 site of YTHDF2 directly binds to and recruits eIF3b to ETV5 mRNA. The current understanding of the mechanism by which YTHDF2 regulates translation is limited and underappreciated, especially compared with the understanding of its canonical role in degrading mRNA. It is necessary to explore whether YTHDF2 participates in translational processes by other molecular mechanisms.

Accumulating evidence has shown that RNA m^6^A modification widely regulates immunological processes.^[^ ^48^ ^]^ It has also been determined that YTHDF2 deficiency in tumor‐associated macrophages suppresses tumor growth by reprogramming tumor‐associated macrophages toward an antitumoral phenotype and increasing their antigen cross‐presentation ability, which in turn enhances CD8^+^ T‐cell‐mediated antitumor immunity.^[^ ^49^ ^]^ Ma et al. demonstrated that depletion of *Ythdf2* in mouse natural killer (NK) cells significantly impaired NK cell antitumor and antiviral immunity. Moreover, YTHDF2 controls NK cell homeostasis, maturation, and survival at a steady–state.^[^ ^50^ ^]^ Depletion of *Ythdf2* in murine myeloid‐derived suppressor cells significantly enhances the suppressive function of these cells by modulating the degradation of Rxra, thus attenuating ConA‐induced liver injury.^[^ ^51^ ^]^ We showed that YTHDF2 promoted HCC immune evasion by upregulating ETV5, which induced the transcription of PD‐L1. In addition, overexpression of YTHDF2 reduced the infiltration of CD8^+^ T cells in the tumor immune microenvironment in HCC. Previous studies indicated that YTHDF2 stabilizes VEGFA transcripts in glioblastoma stem cells in an m^6^A‐dependent manner.^[^ ^11^ ^]^ The m^6^A site at IRES‐A suppresses uORF impairment while facilitating G‐quadruplex‐induced translation of VEGFA in lung cancers.^[^ ^52^ ^]^ In the current study, we found that the mRNA expression of VEGFA was upregulated by YTHDF2 via ETV5 in HCC. These results indicate that the network by which YTHDF2 regulates the expression of VEGFA in different tumor cell backgrounds is complex.

Combination therapy (antiangiogenic antibodies and immune checkpoint inhibitors) exerts superior effects compared with single therapy for HCC treatment.^[^ ^2^, ^21^ ^]^ Kohei Shigeta et al. demonstrated that anti‐VEGFR‐2 blockade in endothelial cells increases PD‐L1 expression in HCC cells and PD‐1 expression in tumor‐infiltrating CD4^+^ cells. After anti‐PD‐1 treatment, CD4^+^ cells promote normalized vessel formation in the context of antiangiogenic therapy with anti‐VEGFR‐2 antibody.^[^ ^21^ ^]^ Similarly, in prostate cancer, inhibition of the binding between VEGF and neurophilin‐2 diminishes PD‐L1 expression and enhances antitumor immunity.^[^ ^53^ ^]^ In the current study, we revealed that YTHDF2 promoted immune evasion and angiogenesis by upregulating PD‐L1 and VEGFA expression. Moreover, targeting YTHDF2 by applying A/Lipo/si‐YTHDF2 complexes significantly inhibited HCC progression. It remains unclear whether the immune evasive effects crosstalk with the angiogenic effects of YTHDF2 or not. Moreover, except for PD‐L1 and VEGFA, our study found that YTHDF2 modulates many crucial genes involved in cancer‐associated signaling pathways (data not shown), implying critical role of YTHDF2 in HCC progression and calling for further researches. Nevertheless, these results provide basic evidence supporting the use of YTHDF2 as an effective therapeutic target for HCC. Besides, we observed that A/lipo/si‐YTHDF2 therapy has less damage to mice normal liver tissue than combined therapy (data not shown). Current developments of small molecule inhibitor (DC‐Y13‐27) targeting YTHDF2 enhanced responses to radiotherapy and immunotherapy,^[^ ^13^ ^]^ suggesting that targeting YTHDF2 may offer novel combination therapeutic strategies for HCC treatment.

## 4 Experimental Section

### Patient Tissue Samples

All human liver tissues were collected from the Hepatic Surgery Center, Tongji Hospital, Huazhong University of Science and Technology (HUST), Wuhan, China. Sixty pairs of snap‐frozen HCC tissues (cohort 1) were used for qRT‐PCR and western blotting analyses of the mRNA and protein levels of YTHDF2. A total of 89 HCC specimens and the counterpart normal liver tissues (cohort 2) were used to make a tissue microarray (Service bio, Wuhan, China) and subjected to IHC analysis.

This research was authorized and supervised by the Ethics Committee of Tongji Hospital (TJ‐IRB20210935) and the study was conducted according to the Declaration of Helsinki principles. Written informed consent was obtained from each patient.

### Chromatin Immunoprecipitation (ChIP) Assays

To detect the enrichment of H3K4me3 and H3K27ac in the promoter region of YTHDF2, three pairs of HCC and ANT tissues (from cohort 1) were selected randomly. Then, 50 mg of HCC and ANT tissues were applied for each immunoprecipitation, respectively. Tissues were frozen into liquid nitrogen and broken by mortar pestle grinding. Transferring minced tissue samples (typically 1 mm^3^ or smaller) to a 15 mL conical tube, and used immediately for chromatin preparation using the SimpleChIP Plus Enzymatic Chromatin IP Kit (#9004, Cell Signaling Tech, USA). The lysate was immunoprecipitated with anti‐H3K4me3, anti‐H3K27ac or IgG antibodies (negative control) overnight. The antibodies used for ChIP assays in this study were listed in Table S8 (Supporting Information). Immunoprecipitated DNAs were extracted and analyzed by qRT‐PCR. The 2000 bp upstream of the YTHDF2 promoter were divided into four parts, and the ChIP primer sequences were listed in Table S9 (Supporting Information).

To determine the binding sites of ETV5 in *PD‐L1* and *VEGFA* promoter, ChIP assay was conducted by using a Magna ChIP kit (17‐10085, Millipore, USA). Briefly, 1 × 10^7^ HLF/OE‐ETV5 and vector cells were cross‐linked in 1% formaldehyde for 10 min at room temperature. After washing with PBS, the cells were resuspended in a lysis buffer. Sonication was used to fragment DNA to 400–800 bp. The supernatants were incubated with anti‐ETV5 antibodies or an isotype control IgG and Protein A/G Magnetic beads at 4 °C for 3 h. The immunoprecipitated DNA was retrieved from the beads with a solution buffer.

The qRT‐PCR was used to amplify the corresponding binding site on the promoters. Enrichment score = 100 × 2^[(Input CT‐Input dilution factor)‐IP CT]^, and the results of enrichment fold were normalized to the enrichment score of IgG in each group. The primers for qRT‐PCR are shown in Table S9 (Supporting Information).

### Animal Experiments

C57BL/6 mice bearing loxP sites flanking exon 3 of the *Ythdf2* gene (*Ythdf2* ^fl/f^) were purchased from Cyagen Biosciences (Guangzhou, China), and hepatocyte‐specific *Ythdf2* knockout mice (*Ythdf2* ^LKO^ mice) were generated by crossing *Ythdf2* ^fl/fl^ mice with Alb‐cre expression mice. Mouse genotypes were identified by tail‐snip PCR amplification using the 2×Taq Plus Master Mix II kit (P213‐01, Vazyme, China) The primers were listed in Table S9 (Supporting Information). Mice brain, lung, colon and liver tissue were lysed by RIPA Lysis Buffer (FY‐RE110, Wuhan Feiyue Biotechnology Co., Ltd., China), and specific *Ythdf2* knockdown was checked by western blotting. To induce spontaneous HCC formation, *Ythdf2* ^LKO^ and *Ythdf2* ^fl/fl^ littermates were intraperitoneally injected with 25 mg k^−1^ g DEN (55‐18‐5, Sigma Aldrich, USA) at postnatal day 15, followed by repeated intraperitoneal injections of a mixture of CCl_4_ (CCl_4_: olive oil = 1:4) (32488‐50‐9, Sigma, USA) at 10 mL kg^−1^ for CCl_4_ twice a week. All mice were sacrificed at 8 months of age.

C57BL/6 mice (4 weeks old) were purchased from Shulaibao Biotechnology (Wuhan, China) for the orthotopic HCC model. The indicated 1 × 10^6^ HCC cells were suspended in 40 µL serum‐free DMEM (SH30022.01B, Hyclone, USA) and Matrigel (356 234, BD Biosciences, USA) mixture (DMEM: Matrigel = 1:2), and then orthotopically implanted into the left hepatic lobe of mice.

For the aptamer/liposome (A/Lipo) siRNA treatment animal experiments, the orthotopic HCC mice model was established as mentioned above. At the day 12, A/Lipo si‐NC or A/Lipo si‐YTHDF2 were intravenously administered every 4 days for a total of 4 times (5 nmol siRNA per mice). All mice were sacrificed at the 4th week.

Tumor volume was quantified according to the formula: tumor volume (mm^3^) = width^2^(mm^2^) ×length(mm)/2. Metastasized tumor foci in lungs were quantified in paraffin‐embedded slides under microscopy. All tumor samples were fixed in formalin and paraffin‐embedded for further histological and IHC analysis. All mice were housed in a temperature‐controlled animal facility with a 12 h light/dark cycle. All animal experiments were approved by the Ethics Committee of Tongji Hospital, HUST (TJH‐202109032). The whole procedure was in accordance with the “Guide for the Care and Use of Laboratory Animals” (NIH publication 86‐23 revised 1985).

### Isolation of Polysome Fractionation

Using prepared solutions containing 10%, 20%, 30%, 40%, 50%, and 60% sucrose, begin by adding 2.2 ml of 10% sucrose gradient to the bottom of the thin‐wall ultracentrifuge tube, and underlay each subsequent layer (2.2 mL). Leave gradients at 4 °C overnight to allow the gradient to become linear. The indicated cells were treated with 100 mg mL^−1^ cycloheximide (CHX, C4859, Sigma–Aldrich, USA) for 10 min at 37 °C. Wash the cells three times with ice‐cold PBS supplemented with 100 µg mL^−1^ CHX. Scrape the cells with cell scraper and transfer the cells to a 15‐mL tube to centrifugation for 5 min at 500 g. Disrupt the cell pellet by pipetting 1 mL polysome extraction buffer (100 µg mL^−1^ CHX, 1×protease inhibitors and 40 U mL^−1^ RiboLock RNase inhibitor) and incubate on ice for 10 min. Centrifuge at 12 000 × g for 10 min at 4 °C. Transfer ≈9/10 of the total volume of the supernatant to a fresh tube. The 1 mL of cytoplasmic extract was carefully layered on the top of sucrose gradient and centrifuged at 19 000 g in a Beckman JA‐30‐50 rotor for 90 min at 4 °C. Take 0.5 mL of each sucrose fraction and the ribosome‐nascent chain complex‐RNA were respectively isolated by Trizol reagent (15596‐026, Invitrogen, USA). The cDNA synthesis and qRT‐PCR were performed by HiScript II Q RT SuperMix (R223, Vazyme, China) and ChamQ Universal SYBR qPCR Master Mix (Q711, Vazyme, China). The percentage of mRNA in each fraction = 2^Ct (fraction 1)‐Ct (fraction X)^ × 100/Sum[2^Ct (fraction 1)‐Ct (fraction X)^].

### Statistical Analyses

The Prism 7.0 software (GraphPad Software, La Jolla, CA, USA) or SPSS 22.0 (SPSS, USA) were used for statistical analyses. The results were presented as the mean ± SD. Quantitative data were analyzed by two‐tailed Student *t‐*test and Pearson's correlation test. Categorical data were analyzed by Chi‐square (χ^2^) test or Fisher's exact test. Kaplan‐Meier analysis was performed to assess the survival between subgroups using log‐rank test. A Cox proportional hazards model was used to determine the independent factors of survival and recurrence based on the variables selected in univariate and multivariate analyses. A value of P < 0.05 was considered statistically significant.

## Conflict of Interest

The authors declare no conflict of interest.

## Author Contributions

J.W., L.X., Y.W., and J.L. contributed equally to this work. J.W., Z.H. and J.L. designed this study and drafted the manuscript. J.W., L.X., Y.W. and J.L. performed the experiments, collected and analyzed the experimental data. J.L., Z.C., S.H., W.J., Y.L., H.L., L.C., T.Y., L.G., Z.D., Q.L. and H.L. provided technical or material support. X.C., B.Z., and Z.H. conceptual advice and supervised the study. X.C., Z.H. and B.Z.: study supervision and obtained funding. All authors read and approved the final manuscript.

## Supporting information

Supporting Information

## Data Availability

The data that support the findings of this study are available from the corresponding author upon reasonable request.
