# Supplementary material for: YTHDF2 Is a Therapeutic Target for HCC by Suppressing Immune Evasion and Angiogenesis Through ETV5/PD‐L1/VEGFA Axis
Source: Adv Sci (Weinh). 2024 Jan 21;11(13):2307242. doi: 10.1002/advs.202307242 (PMC10987122; doi:10.1002/advs.202307242)
Supplement: Supplementary file 1 — Supporting Information [file ADVS-11-2307242-s001.pdf]

ADVANCED SCIENCE
Open Access

Supporting Information

for *Adv. Sci.*, DOI 10.1002/advs.202307242

YTHDF2 Is a Therapeutic Target for HCC by Suppressing Immune Evasion and Angiogenesis Through ETV5/PD-L1/VEGFA Axis

Jingyuan Wen, Lin Xue, Yi Wei, Junnan Liang, Wenlong Jia, Tuying Yong, Liang Chu, Han Li, Shenqi Han, Jingyu Liao, Zeyu Chen, Yiyang Liu, Qiumeng Liu, Zeyang Ding, Huifang Liang, Lu Gan, Xiaoping Chen, Zhao Huang* and Bixiang Zhang**

YTHDF2 is a therapeutic target for HCC by suppressing immune evasion and angiogenesis through ETV5/PD-L1/VEGFA axis

Jingyuan Wen[+], *Lin Xue*[+], *Yi Wei*[+], *Junnan Liang*[+], *Wenlong Jia*, *Tuying Yong*, *Liang Chu*, *Han Li*, *Shenqi Han*, *Jingyu Liao*, *Zeyu Chen*, *Yiyang Liu*, *Qiumeng Liu*, *Zeyang Ding*, *Huifang Liang*, *Lu Gan*, *Xiaoping Chen**, *Zhao Huang**, *Bixiang Zhang**

J. Wen, L. Xue, Y. Wei, J. Liang, W. Jia, L. Chu, H. Li, S. Han, J. Liao, Z. Yu, Y. Liu, Q. Liu, Z. Ding, H. Liang, X. Chen, Z. Huang, B. Zhang

Hepatic Surgery Center, Tongji Hospital, Tongji Medical College, Huazhong University of Science and Technology, Wuhan 430030, China

E-mail: chenxpchenxp@163.com; huangzhao@tjh.tjmu.edu.cn; bixiangzhang@163.com

J. Wen, L. Xue, Y. Wei, J. Liang, W. Jia, L. Chu, H. Li, S. Han, J. Liao, Z. Yu, Y. Liu, Q. Liu, Z. Ding, H. Liang, X. Chen, Z. Huang, B. Zhang

Clinical Medical Research Center of Hepatic Surgery at Hubei Province, Wuhan 430030, China

J. Wen, L. Xue, Y. Wei, J. Liang, W. Jia, L. Chu, H. Li, S. Han, J. Liao, Z. Yu, Y. Liu, Q. Liu, Z. Ding, H. Liang, X. Chen, Z. Huang, B. Zhang

Hubei Key Laboratory of Hepato-Pancreatic-Biliary Diseases, Tongji Hospital, Tongji Medical College, Huazhong University of Science and Technology, Wuhan 430030, China

T. Yong, L. Gan

National Engineering Research Center for Nanomedicine College of Life Science and Technology Huazhong University of Science and Technology Wuhan 430074, China

X. Chen, B. Zhang

Key Laboratory of Organ Transplantation, Ministry of Education; Key Laboratory of Organ Transplantation, National Health Commission; Key Laboratory of Organ Transplantation, Chinese Academy of Medical Science, Wuhan 430030, China

[+] JW, LX, YW and JL contributed equally.

Table of contents

Materials and Methods 3

Figure S1. 17

Figure S2 19

Figure S3 19

Figure S4 20

Figure S5 22

Figure S6 24

Figure S7 26

Figure S8 27

Figure S9 27

Figure S10 28

Table S1 29

Table S2 30

Table S3 32

Table S4 33

Table S8 34

Table S9 36

Table S10 39

Table S11 40

References 41

Materials and Methods

In silico analysis

Four transcriptome profiles from Gene Expression Omnibus (GEO) (GSE36376, GSE14520, GSE64041), ICGC-LIRI-JP and TCGA-LIHC dataset were collected to analyze the mRNA level of *YTHDF2* in HCC and normal tissues. For TCGA-LIHC dataset, the overall survival (OS) and recurrence-free survival (RFS) of 369 HCC patients were analyzed by the Kaplan-Meier website (http://kmplot.com/analysis/index.php) after stratifying patients into two groups based on the median level of YTHDF2 mRNA. The public proteomic expression profile (OEP000321) including 159 paired HCC and ANT samples was obtained from Fan's study to evaluate the protein level of YTHDF2 and its correlation with OS and RFS in HCC patients.[1]

The transcriptome and copy number variation (CNV) profiles in TCGA-LIHC and GSE9829 datasets were obtained from the UCSC Xena (http://xena.ucsc.edu/) and GEO datasets. Amplification or deletion alterations among the whole genome were further identified using GISTIC 2.0 software.[2] Pearson's correlation analyses were conducted between gene expression and CNV value in the GSE9829 dataset to examine the regulation of YTHDF2 expression by CNV.

The publicly available datasets of H3K4me3 and H3K27ac occupancy were accessed from the UCSC/ENCODE browser (http://genome.ucsc.edu/ENCODE/).

The single-cell RNA-seq data was downloaded from GSE202642,[3] *Seurat* package (v.4.3.0) was applied to the processing of the gene expression matrix.[4] The expression data were normalized using the *NormalizeData* function with default parameters. The *FindVariableFeatures* function with the vst method was used to filter the top 3000 variable genes. The principal component analysis (PCA) and Uniform Manifold Approximation and Projection (UMAP) analysis were performed for dimension reduction of gene expression. To infer cell-cell communication, the *CellPhoneDB* package was adopted to explore the ligand-receptor pairs between niche cell subtypes and malignant cells.[5] The interaction between distinct cell subpopulations via putative ligand-receptor pairs was visualized using *pheatmap* package.

Pearson correlation coefficients between mRNA expression of YTHDF2 and all protein-coding genes were calculated and the genes correlated with YTHDF2 expression in the TCGA-LIHC dataset were identified ($p<0.05$, $R\geq0.3$ or ≤-0.3). Further KEGG pathway enrichment analysis was performed using the *clusterProfiler* R package. GO pathway enrichment of RIP-seq genes (Table S6) was also implemented by the *GO seq* R package.

The promoter region (-2000 to -1 bp) of PD-L1 or VEGFA were amplified to predict transcription factors using the JASPAR database (http://jaspar.genereg.net), respectively. ETV5 binding sites on PD-L1 and VEGFA promoters were also predicted using the JASPAR database.

Three-dimensional crystal structures of YTHDF2 and eIF3b protein were predicted using online tools ALPHAFOLD (https://cosmic-cryoem.org/tools/alphafold/). Then, these crystal structures were subjected to protein preprocess, regenerated states of native ligand, H-bond assignment optimization, protein energy, minimization and remove waters by applying the Protein

Preparation Wizard module in Schrödinger Maestro 13.5. Next, molecular docking between YTHDF2 and eIF3b was performed using Schrödinger Maestro 13.5 (Number of ligand rotations to probe=70,000 and Maximum poses to return=30). Additionally, the Protein Interaction Analysis was utilized to determine the specific region of eIF3b-4 that interacts with the N-terminal of YTHD2.

Immunohistochemistry staining (IHC) and analysis

IHC analyses were performed as previously described.[6] The universal SP kit (SP-9000, ZSGB-BIO, China) and DAB substrate (ZLI-9019, ZSGB-BIO, China) were used according to the manufactures' protocol. The antibodies used for IHC in this study were listed in Table S8. The staining intensity of the entire section and the protein expression levels were independently scored by three pathologists. The staining intensity of YTHDF2, ETV5, PD-L1 and VEGFA was scored as 0 (negative), 1 (weak), 2 (moderate), or 3 (strong). The degree of staining was scored based on the percentage of positive cells as follows: 0 (0%), 1 (1-25%), 2 (26-50%), 3 (51-75%), and 4 (76-100%). The staining intensity and degree scores were multiplied to determine the final score for each sample. Ki67 positive rate was analyzed with Image J software.

Western blot analysis

Western blot analysis and quantification of optical densities were performed as described previously[6]. The antibodies used for western blot in this study were listed in Table S8.

RNA isolation and qRT-PCR analysis

Total RNA was extracted using the TRIzol reagent (15596026CN, Invitrogen, USA) according

to the manufacturer's instructions. HiScript® II Q RT SuperMix (R223, Vazyme, China) was used for reverse transcribing cDNA. qRT-PCR was performed using ChamQ Universal SYBR qPCR Master Mix (Q711, Vazyme, China) according to manufacturers' introductions. In cohort 1, the relative YTHDF2 mRNA levels in each sample were detected and compared with the NO.1 sample by comparative threshold cycle ($2^{-\Delta CT}$). In cell lines, relative RNA expression levels were determined by comparative threshold cycle ($2^{-\Delta\Delta CT}$) and each experiment was independently repeated three times at least. Primers used in the study have been listed in Table S9.

Cell lines and cell culture

HepG2, Hep3B, Huh7, Hepa1-6, HUVEC and human embryonic kidney cells 293T (HEK-293T) were purchased from China Center for Type Culture Collection (CCTCC, Wuhan, China). HUVEC cells in passages 2-7 were used. HCC cell lines MHCC97H and HCCLM3 were obtained from the Liver Cancer Institute (Shanghai, China). HCC cell line PLC/PRF/5 was purchased from the cell bank of the Chinese Academy of Sciences (Shanghai, China). HLF was purchased from CoBioer (Nanjing, China). The murine AML12 hepatocyte cell line was obtained from the American Type Culture Collection. All cells were cultured by Dulbecco's Modified Eagle Medium (DMEM) supplemented with 10% fetal bovine serum (FBS) (10099-141, Gibico, USA) at 37 °C in a 5% CO_2 incubator. HCC cell lines used in this study were authenticated by short-tandem repeat profiling at CCTCC.

Enzyme-linked immunosorbent assay (ELISA)

The supernatant of indicated HCC cells was collected and centrifuged at 2000 × g for 3 min. The supernatant was filtered with a 0.22 µm filter to deplete any cell debris and used immediately

for subsequent experiments. The levels of the secreted form of VEGFA (sVEGFA) in the indicated cell supernatants were measured by ELISA kits (RX202521M and RX105007H, Ruixinbio, China) according to the manufacturer's instructions. The absorbance was read at 450 nm by Universal Microplate Reader ELx 800 (Bio-Tek, USA).

***In vitro* T cell mediated cytotoxicity assay**

The human $CD8^+$ T cells were isolated from the peripheral blood of healthy humans by EasySep™ Release Human CD8 Positive Selection Kit (#18053, STEMCELL Technologies, Canada). Isolated human $CD8^+$ T cells were maintained in TexMACS™ GMP Medium (170-076-307, Miltenyi Biotec, China) and 300 U/mL Interleukin-2 (200-02, Peprotech, USA) in the pre-coated plate with 5 μg/mL anti-CD3 (300301, BioLegend, USA) and 5 μg/mL anti-CD28 (302901, BioLegend, USA). The mice $CD8^+$ T cells were isolated from C57BL/6 spleen by using Mouse $CD8^+$ T cell isolation kit (B90011, Selleck, Shanghai, China) according to the manufacturer's protocol. Isolated mice $CD8^+$ T cells were maintained in complete RPMI 1640 medium (10% FBS, 20 Mm HEPES, 1 mM sodium pyruvate, 0.05 mM 2-mercaptoethanol, 2 mM L-glutamine and 50 U/ml streptomycin and penicillin) with 5 ng/mL Interleukin-2 (90142ES08, YEASEN, China), 2 μg/mL anti-CD28 (A2108, Selleck, China) and 2 μg/mL anti-CD3ε (A2104, Selleck, China). The $CD8^+$ T cells (effector cells, E) were cocultured with IFN-γ (300-02-20 or 315-05-20, PeproTech, USA) pre-treated HCC cells (target cells, T) at the indicated ratio for 24 h. The apoptosis rate of tumor cells was detected by Annexin V-PE Apoptosis Detection Kit (A211-01, Vazyme, China).

***In vitro* angiogenesis assay**

The indicated 1×10^7 HCC cells were seeded in 6-well plates, After the cells became adherent,

the medium was replaced with DMEM without FBS. The supernatant medium that cultured HCC cells for 48 h was collected as tumor-conditioned medium (TCM), which contained secreted factors from HCC cells. The TCM was filtered with a 0.22 μm filter, and either used immediately or stored at -80°C for subsequent *in vitro* angiogenesis assay. Tips and 24-well plate were placed on ice for 20-30 min before loading the plate so that the Matrigel (356234, BD Biosciences, USA) did not prematurely solidify. Place the Matrigel in 4 °C the day before use, allowing the Matrigel slowly thaw overnight. HUVEC cells were starved in serum-free medium for 24 h in advance. After 400 μL of diluted Matrigel (Matrigel: DMEM=1:1) was transferred to a 24-well plate on ice, the plate was put into incubator at 37 °C and 5% CO_2 for 3 h to solidify Matrigel. HUVECs at 4×10^4 cells/well were seeded in the Matrigel-coated 24-well plate with 1 mL of various TCM. The cells were observed under a microscope to detect the formation of capillary-like structures after incubating for 6 h at 37 °C, and the results of tuber and branch points were quantified by Image J software.

RNA immunoprecipitation (RIP), m^6A specific methylated RNA immunoprecipitation (meRIP) and sequencing

The RIP and meRIP assay were performed by RNA-Binding Protein Immunoprecipitation Kit (17-700, Millipore, USA) according to the manufacturer's instructions. Briefly, 6×10^7 HCC cells treated with or without 10 μM 3-deazaadenosine (DAA, D8296, Sigma Aldrich, USA) for 24 h were lysed by Lysis buffer at 4 °C for 10 min. The lysate was centrifuged at 12,000×g for 10 min, and 10% supernatant was collected as input. Primary antibodies (YTHDF2 and m^6A) or IgG conjugated prewashed Protein G Magnetic Beads were used to incubate the remaining supernatant in lysis buffer supplemented at 4 °C overnight. Then, wash the Magnetic Beads a total of six times

with cold Wash Buffer, and re-suspend each immunoprecipitated in 150 μL proteinase K buffer. After incubating all tubes at 55 °C for 30 min with shaking, centrifuge the tubes briefly and place the tubes on the magnetic separator. Transfer the supernatant into a new tube to purify RNA. Finally, RNA was further re-suspended in 20 μL of RNase-free water and detected by qRT-PCR with the normalization of Input. Three independent biological replicates were performed for RIP-seq and meRIP-seq. Below are the formulas used for the calculation: ΔCt (normalized RIP) =Ct (RIP) – [Ct (Input)-Log_2 (Input Dilution Factor)]; ΔΔCt (RIP/IgG) =ΔCt (normalized RIP)-Ct (normalized IgG); Fold Enrichment = $2^{(-\Delta\Delta Ct\ [RIP/IgG])}$. The antibodies used for RIP and meRIP assays in this study were listed in Table S8.

The anti-YTHDF2 RIP samples and eluted m^6A-containing fragments in MHCC97H were used for library construction by Lc-Bio Technologies (Hangzhou, China). The total RNA quality and quantity were analyzed by Bioanalyzer 2100 and RNA 6000 Nano LabChip kit (Aglent, CA, USA) with RIN number>7.0. Approximately more than 20 μg of total RNA was subjected to isolate Poly (A) mRNA with poly-T oligo attached magnetic beads (Invitrogen). Following purification, the poly (A) mRNA fractions is fragmented into ~100-nt-long oligonucleotides using divalent cations under elevated temperature. Then the cleaved RNA fragments were subjected to incubation for 2 h at 4 °C with m^6A-specific antibody in IP buffer (50 mM Tis-HCl, 750 mM NaCl and 0.5% Igepal CA-630) supplemented with BSA (0.5 μg/μL). The mixture was then incubated with protein-A beads and eluted with elution buffer (1×IP buffer and 6.7 mM m^6A). Eluted RNA was precipitated by 75% ethanol. Eluted m^6A-containint fragments (IP) and untreated Input control fragments are converted to final DNA library in accordance with a strand-specific library preparation by the dUTP method. The average insert size for the paired-end libraries was ~100±50 bp. Then we performed the paired-end 2×150 bp sequencing on an Illumina Novaseq™ 6000

platform following the vendor's recommended protocol.

mRNA stability assay

To measure mRNA stability in YTHDF2 stable knockdown or control MHCC97H cells, actinomycin D (S8964, Selleck, United States) at 5 μg/mL was added to cells, and the cells were collected after incubation at the indicated times (0, 3, 6 and 9 h). RNA was then extracted for qRT-PCR analysis. The qRT-PCR was performed to evaluate the remained target mRNA level. HRPT1 was used as endogenous control. The date of each group was normalized to the value of the 0 h sample. Primer sequences are listed in Table S9.

Co-immunoprecipitation (co-IP)

Cell samples were lysed with IP lysis buffer (20 mM Tris-HCl, pH 8.0, 137 mM NaCl, 1% NP-40, 2 mM EDTA and 10% glycerol) and centrifuged at 12,000×g for 15 min at 4 °C. The protein supernatant was precleared with Protein G agarose beads (37478S, Cell Signaling Technology, USA) for 2 h at 4°C. The indicated antibodies (Table S8) were added to the protein supernatant and incubated overnight at 4°C. The next day, the antibody-supernatant complex was incubated with prewashed Protein G agarose beads for 3 h at 4°C. The beads were washed twice with IP lysis buffer and 5 times with IP wash buffer. Beads were then boiled with loading buffer to produce the IP products for subsequent western blot analysis.

Dual-luciferase reporter assay and MS2 tethering assays

The whole sequence (2000 bp upstream of TSS.), truncated or mutant promoter sequences of PD-L1 and VEGFA were cloned into the pGL4.17 vector (E6721, Promega, USA). The HLF/OE-

YTHDF2 or vector HLF cells were seeded into 24-well plates at a confluence of approximately 60%. After 12 h, 200 ng of the pGL4.17 plasmid and 4 ng of the phRL-TK-Renilla-luciferase (E6241, Promega, USA) plasmid were co-transfected into each well. The total cell was extracted with Passive Lysis Buffer (E1941, Promega, USA), and luciferase activity was determined using the Dual-Luciferase® Reporter Assay System (E1910, Promega, USA) with a GloMax 20/20 Luminometer (Promega, USA). FLuc luciferase values were normalized against RLuc luciferase activity, and the ratio of FLuc/RLuc luciferase activity is presented. Each experiment was repeated three times.

For the tethering assay, the 5'UTR and 3'UTR of ETV5 was cloned into a firefly luciferase reporter pGL4.17-FLuc-MS2bs (Promega, USA) with MS2 binding sites. The 500 ng of the plasmid expressing MS2-YTHDF2/MS2-YTHDF2-mut/MS2-YTHDF2-W278A, 500 ng of the pGL4.17-FLuc-MS2bs and 10 ng of the pRL-TK-Renilla-luciferase were co-transfected into 12-well plates. After 48 h, half of cells were lysed by 200 μL 1×Passive Lysis Buffer (E1941, Promega, USA). Subsequently, the lysates were treated with the Dual-Luciferase® Reporter Assay System (E1910, Promega, USA) to measure the activities of firefly luciferase and Renilla luciferase on a GloMax 20/20 luminometer (Promega, USA). The remaining cells were treated with TRIzol reagent (15596026CN, Invitrogen, USA), and the mRNA expression was determined by qRT-PCR. Firefly luciferase (FLuc) activity was measured and normalized to the Renilla luciferase (RLuc) activity. Relative FLuc activity was normalized to the relative FLuc-MS2bs mRNAs level.

GST pull-down and His-pulldown assay

The YTHDF2 coding sequence was cloned into the pGEX-6p-1-GST vector. The eIF3 family members' coding sequences were cloned into the pET-28a-HIS vector, respectively. These plasmids were transformed into BL21 Escherichia coli (C504-02, Vazyme, China), and the expression of recombinant proteins were induced with 1 mM IPTG (420322, Millipore, USA) at 20 °C overnight. The bacteria were pelleted and resuspended in PBS-T buffer containing protease inhibitors (P8340, Sigma-Aldrich, USA) and then lysed by sonication. The recombinant proteins were purified by the BeaverBeads IDA-Nickel kit (70501-5, Beaver, Suzhou, China) or the GST Purification Magbeads (#abs9902, Absin, China). 5 μg GST-YTHDF2 was incubated with or without 5 μg His-eIF3 protein in PBS at 4 °C for 4 h under constant mixing. The bound proteins were incubated and immunoprecipitated with anti-GST Magnetic Beads (HY-K0222, MedChemExpress, USA) or anti-His Magnetic Beads (HY-K0209, MedChemExpress, USA) for 2 h at 4 °C. The beads were then extensively washed, and the fusion proteins were resuspended in 30 μL loading buffer (#1610747, Bio-Rad, USA) for subsequent western blotting analysis. Silver staining was performed with the silver stain kit (P0017S, Beyotime, Shanghai, China) following the manufacturer’s recommendations.

Immunofluorescence staining and confocal analysis for colocalization

Immunofluorescence assays were performed as described previously[7]. Briefly, cells were cultured on coverslips for 12 h, fixed in 4% paraformaldehyde for 15 min at room temperature, and permeabilized with 0.5% Triton X-100 for 5 min. After blocking, slides were incubated with anti-YTHDF2 and anti-eIF3b (Table S8) overnight at 4 °C. The slides were then washed thrice and incubated with secondary antibody for 4 h at room temperature. Finally, the cell nuclei were stained with DAPI (RM02978, Abclonal, China) for 5 min. The resulting signals were visualized using a

confocal laser scanning microscope (Olympus FV1000, Tokyo, Japan). The relative fluorescence intensity was analyzed and quantified using the Image-Pro Plus 6.0 image software.

Flow cytometry analysis

HCC cell lines were treated with 20 ng/mL IFN-γ (300-02-20 or 315-05-20, PeproTech, USA) for 24 h. Treated cells were collected and washed three times with PBS, and then suspended in 200 μL of PBS. Anti-PD-L1 antibodies were added into cell suspension (1:50) for 30 min at room temperature. After washing with PBS three times, stained cells were resuspended with 300 μL PBS for flow cytometry analysis.

Liver tumors were digested with 20 mg/mL DNase I (D4513, Sigma Aldrich, USA) and 1.5 mg/mL collagenase (C5138, Sigma Aldrich, USA) for 30 min at 37°C. Then, tumor masses were grinded into single cell suspension and filtered by 70 μm-pore filters (BS-70-XBS, Biosharp, China). To detect the proportion of immune cells, the leukocytes were isolated by a Mouse Lymphocyte Isolation Kit (P9000, Solarbio, China) according to the manufacturer's instructions. The cell pellet was resuspended with PBS containing 2% FBS and incubated with the indicated primary antibodies (Table S10) for at least 1 h at 4°C in the dark. The cells were fixed with 1:1 mixture of 4% paraformaldehyde/PBS-2% FBS and medium for 1 hour and were centrifuged at 1,600 rpm for 5 min at room temperature. Then the cell pellets were resuspended with PBS-2% FBS for flow cytometry analysis. Data acquisition was done on a FACS Fortessa (BD Biosciences) and analysis was performed using FlowJo.

Plasmid constructs and lentivirus construction

The Flag-YTHDF2 (1-579 amino acids), N-YTHDF2 (1-384 amino acids), C-YTHDF2 (385-

579 amino acids) and YTHDF2-mut (K416A, W432A, W486A, W491A and R527A) mutant plasmid[8] was kindly gifted by Professor Tiebang Kang at Sun Yat-sen University Cancer Center. The above sequence were cloned into vector pcDNA3.1(-)-3FN by homologous recombination using ClonExpress II One Step Cloning Kit (C112-01/02, Vazyme, China). The sequence of MS2 and the full-length coding sequence of MS2-YTHDF2 were also cloned into the pcDNA3.1-3FN vector. Plasmid encoding eIF3b was purchased from Wuhan Biophull Biotechnology Co., LTD, and the coding sequence of eIF3b was cloned into vector pcDNA3.1(+)-HAC by ClonExpress II One Step Cloning Kit (C112-01/02, Vazyme, China). The five truncated sequences of eIF3b (eIF3b-1, eIF3b-2, eIF3b-3, eIF3b-4 and eIF3b-5) were amplified from pcDNA3.1(+)-HAC-eIF3b by ClonExpress II One Step Cloning Kit (C112-01/02, Vazyme, China). The pcDNA3.1(-)-3FN-MS2-YTHDF2-mut (K416A, W432A, W486A, W491A, R527A), pcDNA3.1(-)-3FN-MS2-YTHDF2-W278A, pcDNA3.1(+)-HAC-eIF3b-H593A, pcDNA3.1(+)-HAC-eIF3b-E601A and pcDNA3.1(+)-HAC-eIF3b-H593A/E601A plasmid were constructed by Mut Express II Fast Mutagenesis Kit V2 Kit (C214-01/02, Vazyme, China).

Sequences of short hairpin RNAs (shRNAs) against YTHDF2 were designed by the GPP portal website (https://portals.broadinstitute.org/gpp/public/) and negative control was referenced from scramble shRNA (#1864, Addgene, USA) (Table S11). The sequences of shRNAs or scramble were separately cloned to pLKO.1-TRC control (#10879, Addgene, USA). Recombinant lentivirus was generated by transient co-transfection of HEK-293T cells with lentiviral transducing vector, and packaging vectors pMD2.G (#12259, Addgene, USA) and psPAX2 (#12260, Addgene, USA) using Lipofectamine 3000 transfection Reagent (L3000075, Thermo Fisher Scientific, USA). After 48 h, the viral supernatant was filtered by a 0.22 μm filter (PALL, Port Washington, USA). YTHDF2 overexpression lentivirus was purchased from DesignGene Biotechnology (Shanghai,

China) based on the pLvx-puro vector. ETV5 overexpression and knockdown lentiviruses were purchased from Hanheng Biotechnology (Shanghai, China) based on pHBLV-CMV-MCS-EF1-Luc-T2A-BSD and pHBLV-U6-MCS-EF1-Luc-T2A-BSD vector respectively. The stable transduced cells were selected by 3 μg/mL puromycin (P8230, Solarbio, China) or 4 μg/mL blasticidin (B9300, Solarbio, China) for 2 weeks.

Cell transfection and reagents

Plasmids were transfected into cells by Lipofectamine 3000 (L3000150, Invitrogen, USA) according to the manufacturer's instructions. The indicated siRNAs were transiently transfected into cells by Lipofectamine RNAi Max (13778150, Invitrogen, USA). The sequences of shRNAs and siRNAs used in this study were shown in Table S11.

OICR-9429 (S7833) was purchased from Selleck (China). CBP/p300-IN-12 (HY-132197) and MG132 (HY-13259) were purchased from MedChemExpress (USA). Actinomycin D (S8964) was purchased from Selleck (USA). DAA (D8296) was purchased from Sigma Aldrich (USA).

The siRNA-containing aptamer/liposomes (A/Lipo) formulation

The YTHDF2 siRNA and the HCC-specific aptamer TLS11a reported in our previous research[9] were obtained from Wuhan QijingBiological Technology Co., Ltd. The siRNA sequences are listed in Table S11. To improve the stability of A/Lipo si-NC and A/Lipo si-YTHDF2, all pyrimidine bases (C/U) in both strands of siRNAs were modified by 2′-O-Methyl. The lipids were kindly gifted by Professor Lu Gan and Dr. TuYing Yong at Huazhong University of Science and Technology. The aptamer was mixed with lipid in absolute ethanol and then added to 250 μL siRNA-citrate buffer accompanied by vortex oscillation for 10 s. Afterward, the

formulations were passed through a 10 KD ultrafiltration centrifuge tube (UFC501096, Millipore, USA) for 30 min. The hydrodynamic size and dispersity were characterized by a Zetasizer Nano ZS (Particle Cell affinity assay

A/Lipo affinity assay

An equal number of Hepa1-6 and AML12 cells were seeded in the coverglass-bottom dish one day before the A/Lipo treatment. An equal amount of PKH26 (MX4021, Maokangbio, China) labeled A/Lipo was added to the plate the next day. After 24 h, cells were wash twice with PBS to remove the labeled A/Lipo, then fixed in 4% paraformaldehyde for 10 min, and the cell nuclei were stained with DAPI. Samples then were mounted with mounting buffer, and images were obtained using a Zeiss LSM780 confocal microscope (Zeiss) and analyzed using Zeiss Zen software (Zeiss).Metrix, Germany).

Tumor-bearing C57BL/6 male mice were injected with a treatment-dose amount of DIR (abs45153692, Absin, China) labeled A/Lipo via tail vein. After 24 h, mice were monitored for *in vivo* bio-distribution using the SI Imaging Lago X 3D system (Spectral Instruments Imaging) and then sacrificed for *in vitro* organ imaging.

Supplementary Figures

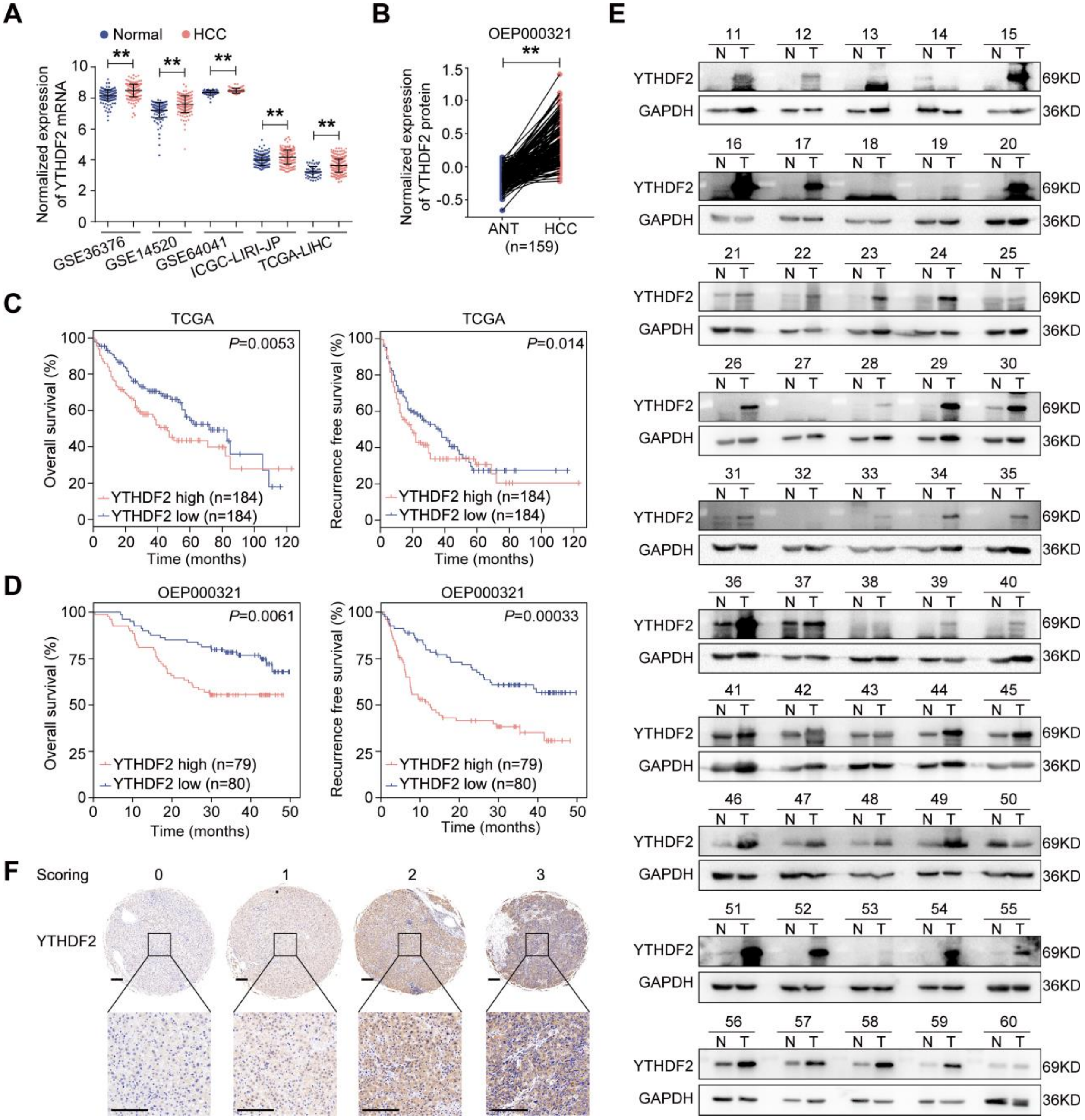

Figure S1. The level of YTHDF2 is higher in HCC compared with normal liver tissues.

(A) Relative mRNA levels of *YTHDF2* in HCC and normal tissues in five independent published patient cohorts (GSE36376: normal n=193, HCC n=240; GSE14520: normal n=151, HCC n=149; GSE64041: normal n=60, HCC n=60; ICGC-LIRI-JP: normal n=177, HCC n=212 and TCGA-

LIHC: normal n=50, HCC n=374). (B) Comparison of YTHDF2 protein expression between matched ANT and HCC tissue (n=159) in OEP000321 dataset. (C and D) HCC patients were divided into two groups (YTHDF2 high or low) according to the median of YTHDF2 mRNA/protein level in TCGA-LIHC (C) or OEP000321 (D), respectively. Kaplan-Meier analyses for overall survival and recurrence-free survival for HCC patients in these two groups. (E) Western blot analysis for YTHDF2 in 50 pairs of HCC samples. GAPDH as loading control. N, adjacent nontumorous tissues; T, tumor. (F) Representative images for IHC score for YTHDF2 staining intensity. Scale bar, 200 μm. Data are shown as the mean ± SD in (A). *, $p < 0.05$; **, $p < 0.01$. Abbreviations: ANT, adjacent nontumorous tissues; HCC, hepatocellular carcinoma; T, tumor; LIHC, Liver hepatocellular carcinoma.

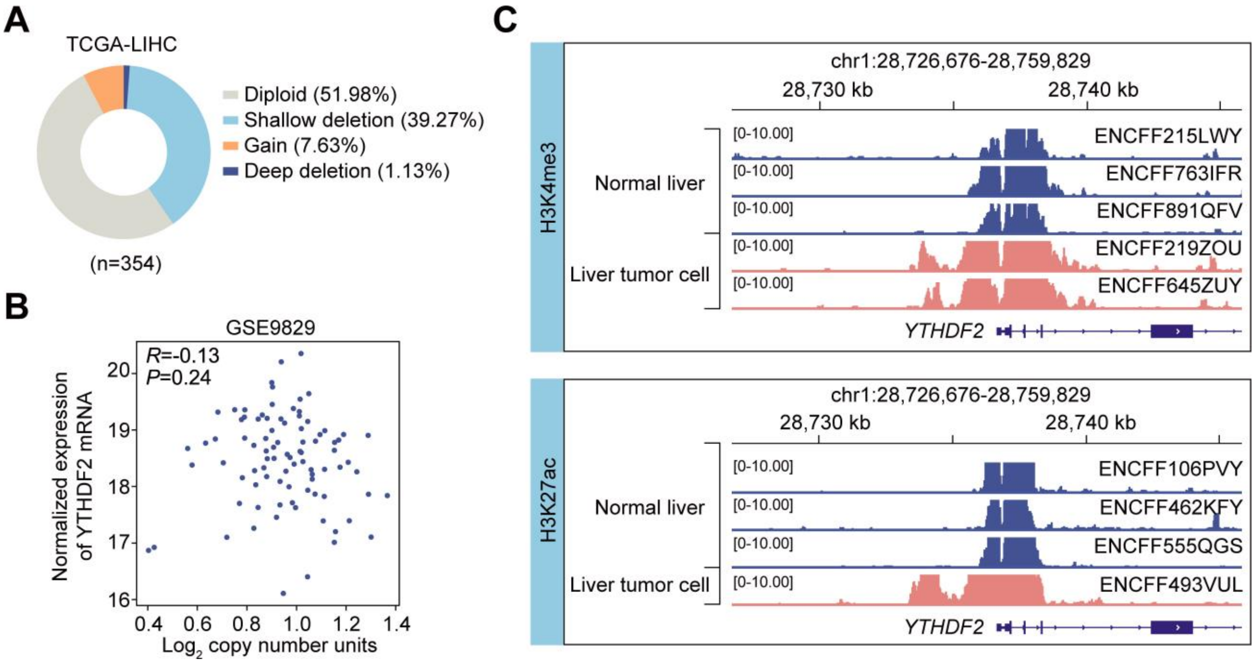

Figure S2. ***In silico*** **analysis of** ***YTHDF2*** **copy number variations and epigenetic modification sites.**

(A) Schematic diagram of the types of CNV found in the *YTHDF2* gene in HCC (data from TCGA-LIHC). (B) Pearson's correlation analyses of the mRNA and CNV levels of the *YTHDF2* gene in HCC samples from a GEO dataset (GSE9829). (C) Analysis of H3K4me3 (upper) and H3K12ac (bottom) in ChIP-seq data (ENCODE) of liver tumor cells and liver tissues at loci upstream of *YTHDF2*. Abbreviations: CNV: copy number variation; H3K4me3, trimethylated histone H3 lysine 4; H3K27ac, H3 lysine 27 acetylation; ChIP, chromatin immunoprecipitation.

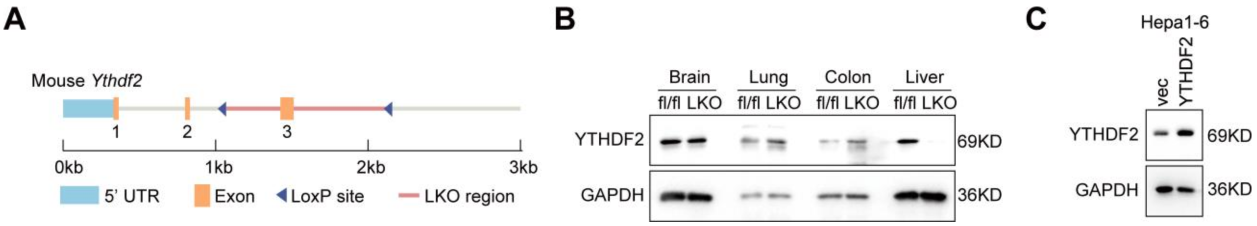

Figure S3. Validation of YTHDF2 knocking down and overexpression efficacy.

(A) Schematic diagram showing *Ythdf2* genome and the strategy for generating conditional *Ythdf2* knockout mice. Exon 3 was flanked by the loxP cassette. (B) Western blot analysis for YTHDF2 expression in different organs of mice with liver specific depletion of *Ythdf2*. (C) YTHDF2 overexpression efficacy in Hepa1-6 cells was examined by western blot. GAPDH as loading control in (B and C). Abbreviations: 5' UTR, 5'-untranslated region; fl/fl, flox/flox; LKO, liver specific knockout; vec, vector.

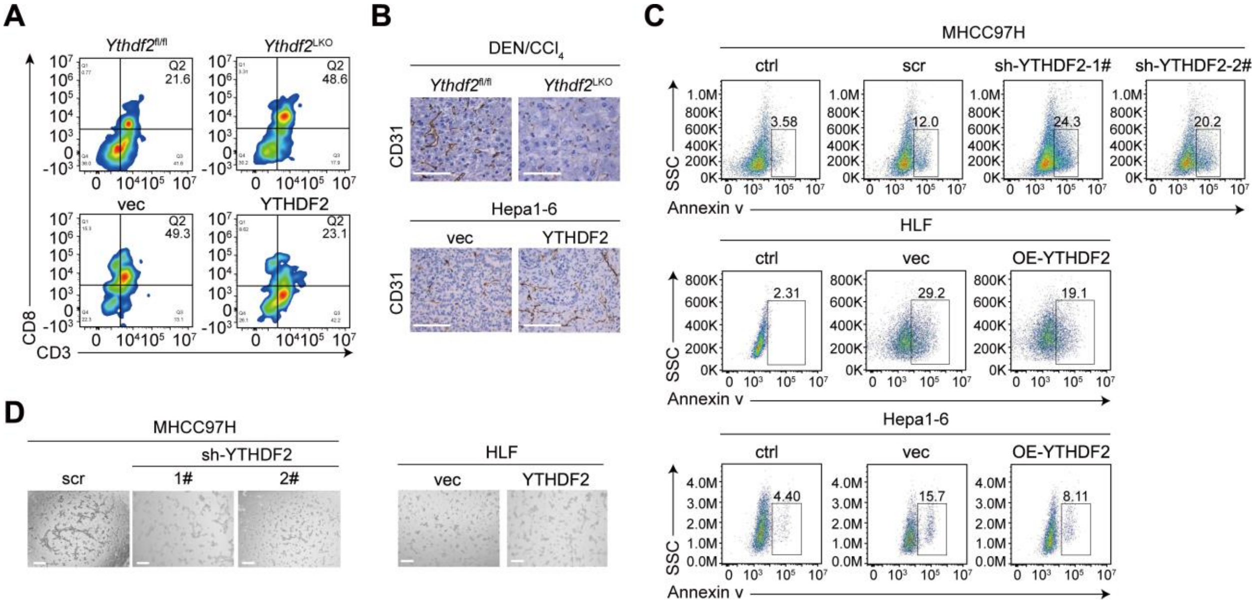

Figure S4. Analysis of the number of CD8$^+$ cells and new vascular tubes.

(A and B) Representative images of flowcytometry analysis of tumor-infiltrating CD8$^+$ T cells (A) and IHC images of CD31 (B) in DEN/CCl_4-induced (*Ythdf2*$^{fl/fl}$, n=5; *Ythdf2*^{LKO}, n=6) and Hepa1-6 xenografted (Hepa1-6-vec, n=7; Hepa1-6-YTHDF2, n=7) liver tumors. Scale bar, 200 μm. (C) Representative flow plots show staining on CD8$^-$ Annexin V$^+$ gated cells. (D) Representative images of HUVEC cell morphology (Scale bar, 100 μm) cultured by different tumor-conditioned medium. Abbreviations: DEN, N-Nitrosodiethylamine; CCl_4: carbon tetrachloride; fl/fl, flox/flox.

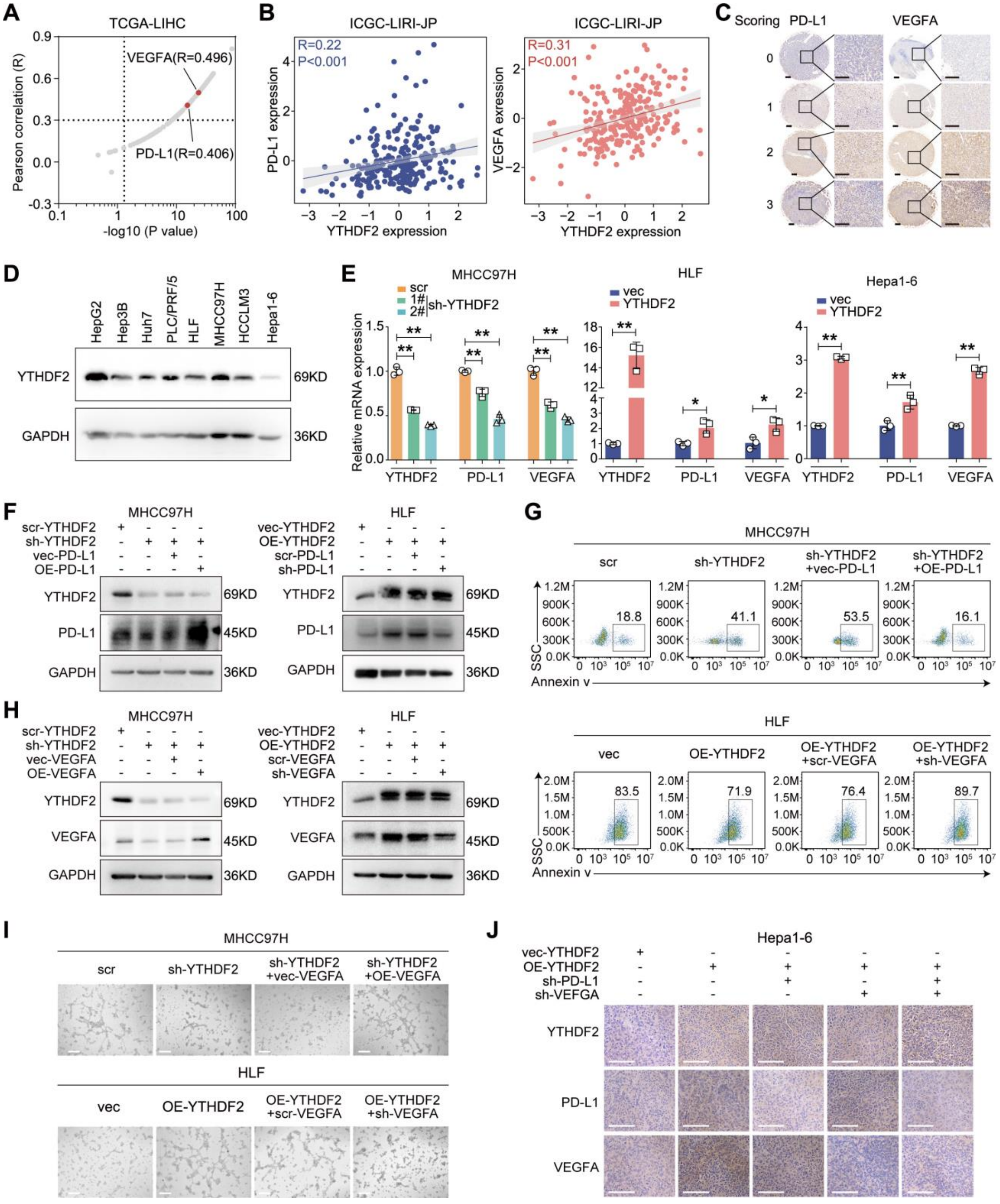
A
TCGA-LIHC
VEGFA(R=0.496)
PD-L1(R=0.406)
Pearson correlation (R)
-log10 (P value)
B
ICGC-LIRI-JP
R=0.22
P<0.001
PD-L1 expression
YTHDF2 expression
ICGC-LIRI-JP
R=0.31
P<0.001
VEGFA exression
YTHDF2 expression
C
Scoring PD-L1 VEGFA
D
HepG2
Hep3B
Huh7
PLC/PRF/5
HLF
MHCC97H
HCCLM3
Hepa1-6
YTHDF2 69KD
GAPDH 36KD
E
MHCC97H
scr
1#
2#
sh-YTHDF2
HLF
vec
YTHDF2
Hepa1-6
vec
YTHDF2
Relative mRNA expression
YTHDF2 PD-L1 VEGFA
F
MHCC97H
scr-YTHDF2
sh-YTHDF2
vec-PD-L1
OE-PD-L1
YTHDF2 69KD
PD-L1 45KD
GAPDH 36KD
HLF
vec-YTHDF2
OE-YTHDF2
scr-PD-L1
sh-PD-L1
G
MHCC97H
scr
sh-YTHDF2
sh-YTHDF2 +vec-PD-L1
sh-YTHDF2 +OE-PD-L1
18.8
41.1
53.5
16.1
SSC
Annexin v
HLF
vec
OE-YTHDF2
OE-YTHDF2 +scr-VEGFA
OE-YTHDF2 +sh-VEGFA
83.5
71.9
76.4
89.7
H
MHCC97H
scr-YTHDF2
sh-YTHDF2
vec-VEGFA
OE-VEGFA
VEGFA 45KD
HLF
vec-YTHDF2
OE-YTHDF2
scr-VEGFA
sh-VEGFA
I
MHCC97H
scr
sh-YTHDF2
sh-YTHDF2 +vec-VEGFA
sh-YTHDF2 +OE-VEGFA
HLF
vec
OE-YTHDF2
OE-YTHDF2 +scr-VEGFA
OE-YTHDF2 +sh-VEGFA
J
Hepa1-6
vec-YTHDF2
OE-YTHDF2
sh-PD-L1
sh-VEFGA
YTHDF2
PD-L1
VEGFA

Figure S5. PD-L1 and VEGFA is the downstream effectors of YTHDF2.

(A) Pearson correlation analysis for the mRNA level of YTHDF2 and the enriched genes in the two KEGG terms ('PD-L1 expression and PD-1 checkpoint pathway in cancer' and 'VEGF signaling pathway') in HCC samples (TCGA-LIHC, n=374). The correlation between YTHDF2 with PD-L1 and VEGFA were spotted. (B) Pearson's correlation analyses of YTHDF2 and PD-L1 or VEGFA in HCC samples from the ICGC-LIRI-JP dataset. (C) Representative images for IHC score for PD-L1 and VEGFA staining intensity. Scale bar, 200 μm. (D) Western blot analysis of YTHDF2 expression in HCC cell lines. GAPDH as loading control. (E) The expression of PD-L1 and VEGFA mRNA were analyzed by qRT-PCR in the indicated HCC cells. HCC cells were treated with IFN-γ (20 ng/mL) for 24 h to detect the expression of PD-L1. Data were normalized to the GAPDH and are shown as fold change relative to their respective control groups (scr or vec). (F) The expression of PD-L1 and YTHDF2 were detected by western blot in YTHDF2 knocked down/overexpressed HCC cells with PD-L1 overexpressed/knocked down. (G) Representative flow plots show staining on $CD8^-$ Annexin V^+ gated cells. (H) The expression of VEGFA and YTHDF2 were detected by western blot in YTHDF2 knocked down/overexpressed HCC cells with VEGFA overexpressed/knocked down. (I) Representative images of HUVEC cell morphology (Scale bar, 100 μm) cultured by different tumor-conditioned medium. (J) Hepa1-6 cells with YTHDF2 stably overexpressed with or without PD-L1/VEGFA knocked down were inoculated in the mice liver (n=7 for each group). Representative images of the IHC staining for the indicated proteins staining for liver tumor (Scale bar, 200 μm). GAPDH as loading control for (D, F and H). Three independent experiments were performed for (E-I). *, $p < 0.05$; **, $p < 0.01$.

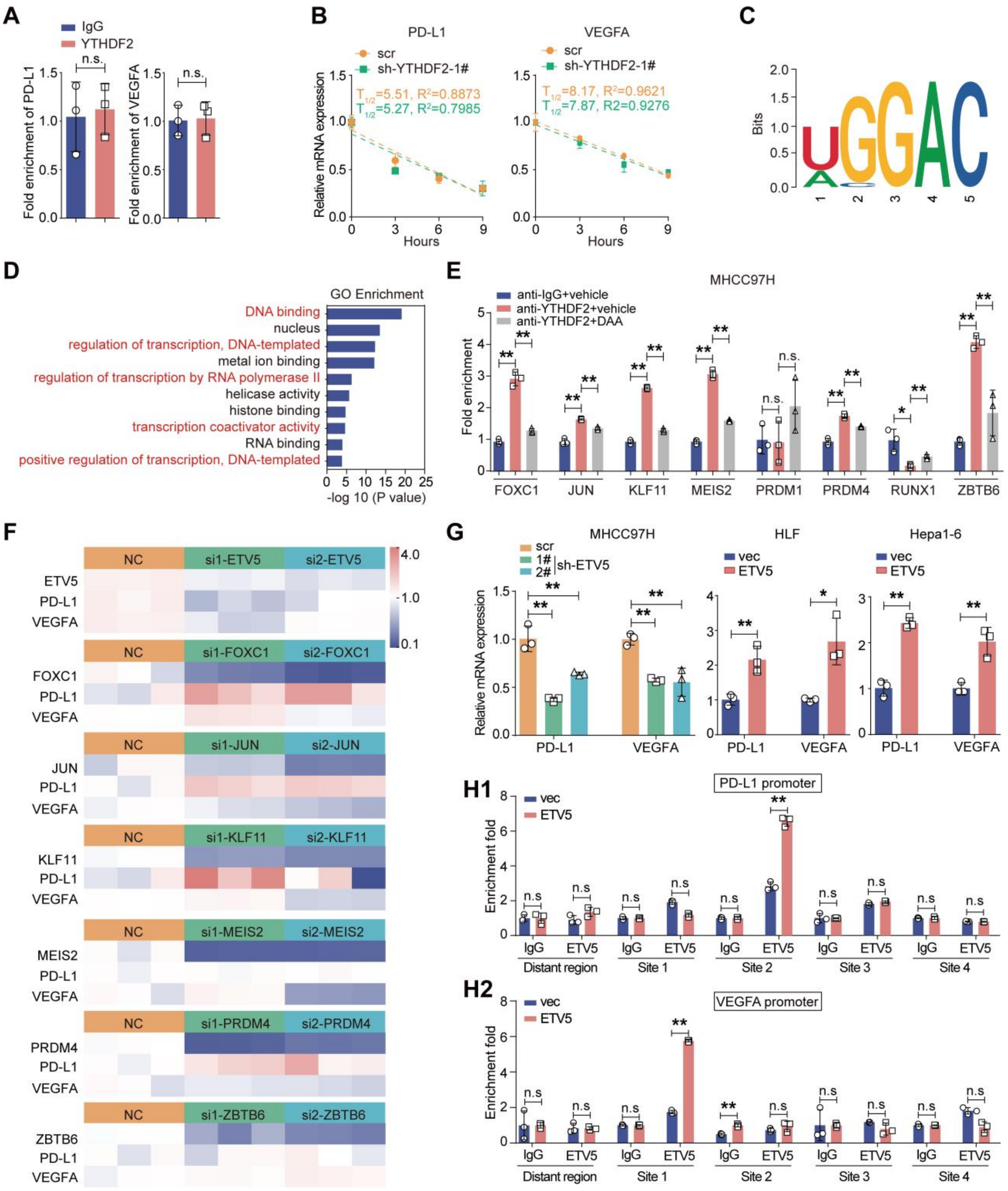
A
IgG
YTHDF2
n.s.
Fold enrichment of PD-L1
Fold enrichment of VEGFA
B
PD-L1
VEGFA
scr
sh-YTHDF2-1#
Relative mRNA expression
Hours
C
Bits
D
GO Enrichment
DNA binding
nucleus
regulation of transcription, DNA-templated
metal ion binding
regulation of transcription by RNA polymerase II
helicase activity
histone binding
transcription coactivator activity
RNA binding
positive regulation of transcription, DNA-templated
-log 10 (P value)
E
MHCC97H
anti-IgG+vehicle
anti-YTHDF2+vehicle
anti-YTHDF2+DAA
Fold enrichment
FOXC1 JUN KLF11 MEIS2 PRDM1 PRDM4 RUNX1 ZBTB6
F
NC si1-ETV5 si2-ETV5
ETV5 PD-L1 VEGFA
NC si1-FOXC1 si2-FOXC1
NC si1-JUN si2-JUN
NC si1-KLF11 si2-KLF11
NC si1-MEIS2 si2-MEIS2
NC si1-PRDM4 si2-PRDM4
NC si1-ZBTB6 si2-ZBTB6
G
MHCC97H HLF Hepa1-6
scr
1# sh-ETV5
2#
vec
ETV5
Relative mRNA expression
H1
PD-L1 promoter
Enrichment fold
Distant region Site 1 Site 2 Site 3 Site 4
H2
VEGFA promoter

Figure S6. ETV5 is the downstream target of YTHDF2.

(A) The qRT-PCR analysis for the PD-L1/VEGFA mRNA level in anti-YTHDF2 RNA precipitates in MHCC97H cells. Data are shown as fold change relative to anti-IgG group. (B) The half-time lifespan of PD-L1 and VEGFA mRNA were analyzed by qRT-PCR in MHCC97H cells with YTHDF2 stably knocked down. Data were normalized to HPRT1 and are shown as fold change relative to 0 h. (C) Sequence motif identified from the m^6A peaks. (D) GO analysis for the enriched genes in RIP-seq. (E) The qRT-PCR analysis for the enrichment of the indicated mRNA in anti-YTHDF2 precipitates in HCC cells with or without DAA treatment. Data are shown as fold change relative to anti-IgG group. (F) HCC cells were transfected with small interference RNA targeting the indicated genes or negative control (NC) for 48 h. qRT-PCR analyses were performed to evaluate the PD-L1 and VEGFA expression. The heatmap exhibited the relative expression of VEGFA and PD-L1 in the indicated HCC cells. (G) The qRT-PCR analysis for the PD-L1 and VEGFA levels in the indicated cells. (H) The direct binding of ETV5 to the putative binding sites on the promoter region of PD-L1 and VEGFA were detected by ChIP assay in ETV5-overexpressed HLF cells. Data are shown as fold changes relative to their respective IgG groups. Data are shown as the mean ± SD in (A, B, E, G and H). Three independent experiments were performed in (A, B and E-H). *, $p < 0.05$; **, $p < 0.01$. Abbreviations: DAA: 3-deazaadenosine.

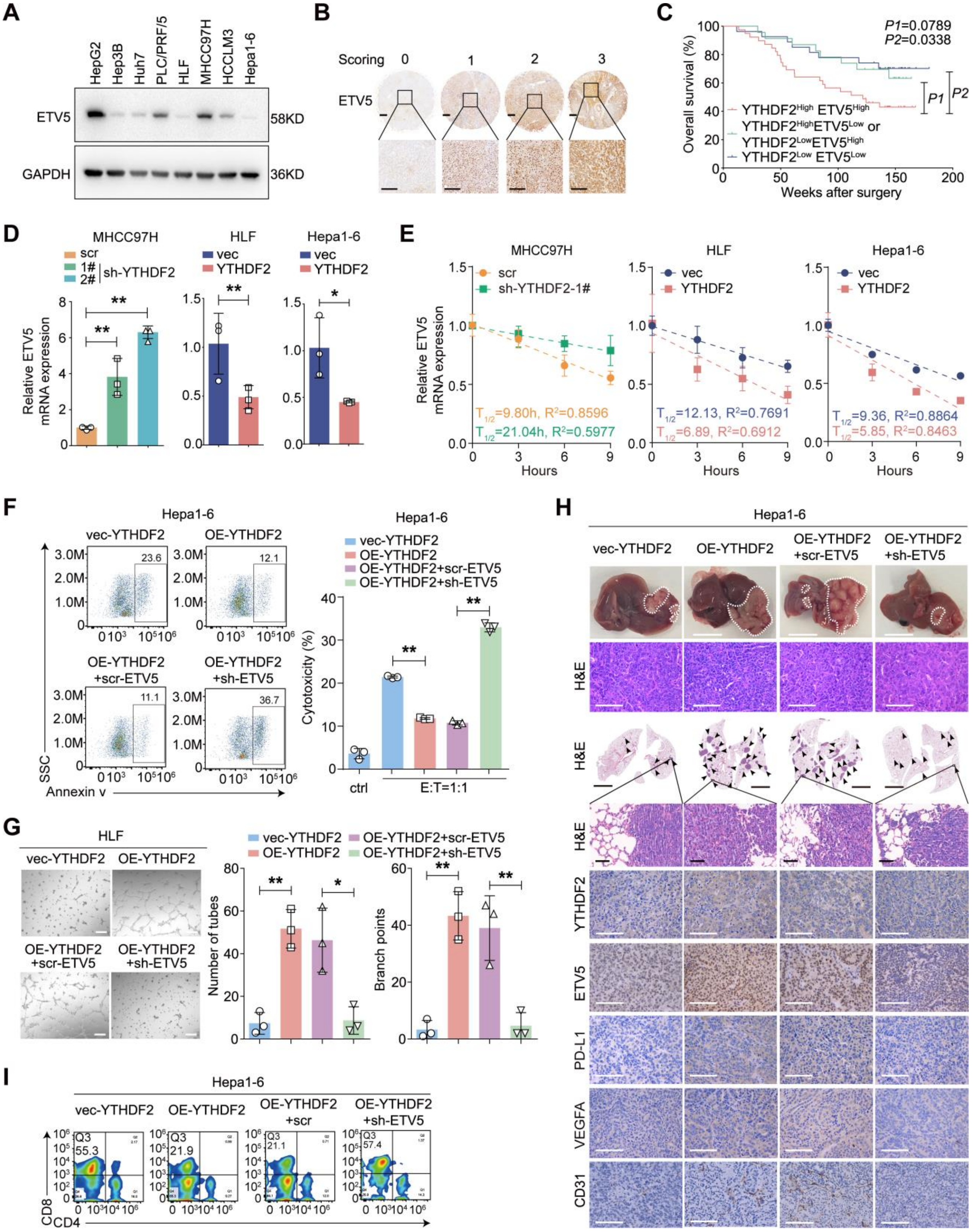
A
HepG2
Hep3B
Huh7
PLC/PRF/5
HLF
MHCC97H
HCCLM3
Hepa1-6
ETV5
58KD
GAPDH
36KD
B
Scoring
0
1
2
3
ETV5
C
Overall survival (%)
Weeks after surgery
P1=0.0789
P2=0.0338
YTHDF2High ETV5High
YTHDF2HighETV5Low or
YTHDF2LowETV5High
YTHDF2Low ETV5Low
D
MHCC97H
HLF
Hepa1-6
scr
1#
2#
sh-YTHDF2
vec
YTHDF2
Relative ETV5 mRNA expression
E
MHCC97H
HLF
Hepa1-6
scr
sh-YTHDF2-1#
vec
YTHDF2
Relative ETV5 mRNA expression
Hours
T1/2=9.80h, R2=0.8596
T1/2=21.04h, R2=0.5977
T1/2=12.13, R2=0.7691
T1/2=6.89, R2=0.6912
T1/2=9.36, R2=0.8864
T1/2=5.85, R2=0.8463
F
Hepa1-6
vec-YTHDF2
OE-YTHDF2
OE-YTHDF2 +scr-ETV5
OE-YTHDF2 +sh-ETV5
23.6
12.1
11.1
36.7
SSC
Annexin v
Hepa1-6
vec-YTHDF2
OE-YTHDF2
OE-YTHDF2+scr-ETV5
OE-YTHDF2+sh-ETV5
Cytotoxicity (%)
ctrl
E:T=1:1
G
HLF
vec-YTHDF2
OE-YTHDF2
OE-YTHDF2 +scr-ETV5
OE-YTHDF2 +sh-ETV5
Number of tubes
Branch points
H
Hepa1-6
vec-YTHDF2
OE-YTHDF2
OE-YTHDF2 +scr-ETV5
OE-YTHDF2 +sh-ETV5
H&E
YTHDF2
ETV5
PD-L1
VEGFA
CD31
I
Hepa1-6
vec-YTHDF2
OE-YTHDF2
OE-YTHDF2 +scr
OE-YTHDF2 +sh-ETV5
Q3
55.3
21.9
21.1
57.4
CD8
CD4

Figure S7. ETV5 mediates the oncogenic effects of YTHDF2.

(A) Western blot analysis for ETV5 expression in the indicated HCC cell lines. GAPDH as loading control. (B) Representative images for IHC score for ETV5 staining intensity. Scale bar, 200 μm. (C) The 89 HCC patients in cohort 2 were stratified into three groups according to the IHC score of YTHDF2 and ETV5. Kaplan-Meier analysis for the overall survival in the three groups. (D) The qRT-PCR analysis for PD-L1 and VEGFA expression in the indicated HCC cells. Data were normalized to GAPDH and are shown as fold change relative to scr or vec group. (E) The half-time lifespan of ETV5 mRNA was analyzed by qRT-PCR in MHCC97H cells with YTHDF2 stably knocked down and HLF/Hepa1-6 cells with YTHDF2 overexpression. Data were normalized to HPRT1 and are shown as fold change relative to 0 h. (F) The indicated HCC cells were co-cultured with $CD8^+$ T cells. The apoptotic rates of HCC cells were detected by flow cytometry. (G) Supernatants from the indicated HCC cells were collected to prepare conditional medium. Tube formation assay was performed by culturing HUVEC cells on matrigel with the indicated tumor conditional medium. The number of tuber and branch points were quantified (Scale bar, 100 μm). (H) Hepa1-6 cells with YTHDF2 stably overexpressed (OE-YTHDF2) with or without ETV5 knocked down (sh-ETV5) were inoculated in the mice liver (n=7 for each group). Representative images of the tumor-bearing liver (Scale bar, 1 cm), H&E and IHC staining for the indicated proteins staining for liver tumor (Scale bar, 200 μm), as well as lung metastases (Scale bar: 2 mm, upper; 50 μm, lower). (I) Flow cytometry analysis for tumor-infiltrating $CD8^+$ T cells in the indicated orthotopic liver tumors. Data are shown as the mean ± SD in (D-G). Three independent experiments were performed for (D-G and I). *, $p < 0.05$; **, $p < 0.01$.

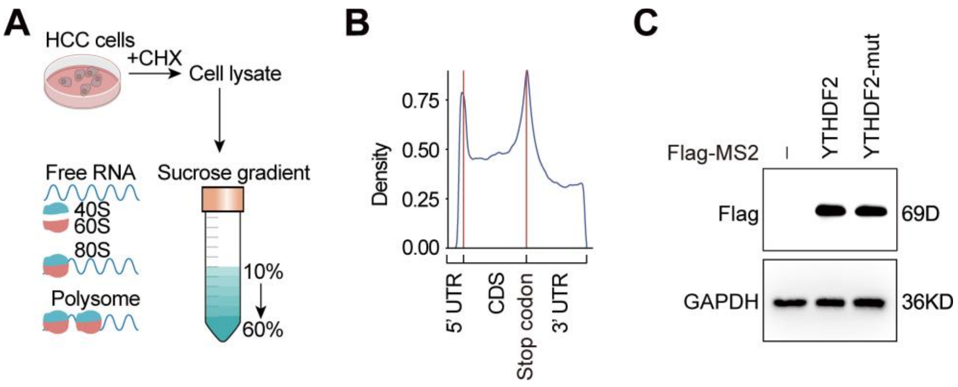

Figure S8. YTHDF2 facilitates ETV5 mRNA translation by recognizing the m^{6}A modification.

(A) Schematic workflow of separation of different ribosome fractions. (B) Distribution of m^{6}A peak reads across all mRNAs in meRIP-seq. (C) Western blot analysis for Flag-MS2 expression in the indicated HCC cells. GAPDH as loading control.

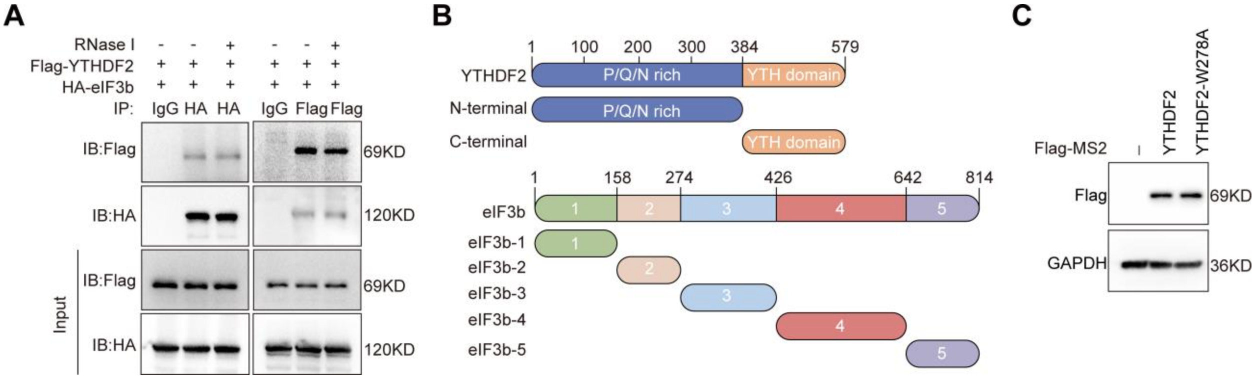

Figure S9. YTHDF2 binds with eIF3b.

(A) Co-IP analysis for the binding between YTHDF2 and eIF3b with or without RNase I treatment. (B) Schematic diagram for wild-type and different truncations of YTHDF2 and eIF3b. (C) Western blot analysis for Flag-MS2 expression in the indicated HCC cells. GAPDH as loading control. Abbreviation: IP, immunoprecipitation; IB, immunoblotting.

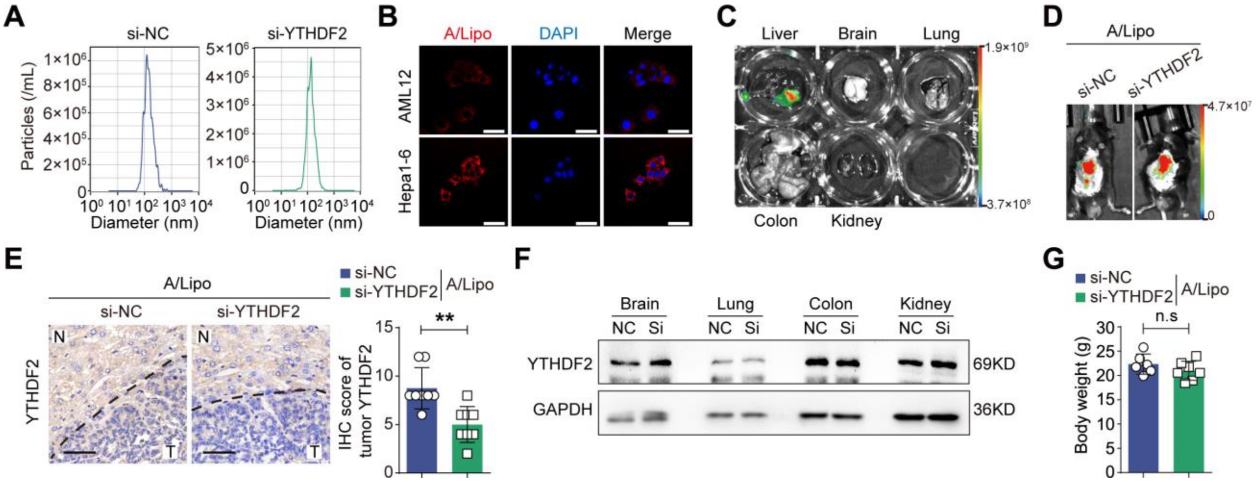

Figure S10. Targeting YTHDF2 by using siRNA-Aptamer/Lipo complexes.

(A) Nanoparticle tracing analysis for the dispersity of siRNA-A/Lipo complexes. (B) Represent images of fluorescent signaling intensity of Hepa1-6 and AML12 cells treated with PKH26-stained A/lipo for 24 hours. Blue, DAPI; red, PKH26. Scale bar, 20 μm. (C) Mice bearing orthotopic Hepa1-6 liver tumors were injected with DiR-stained A/Lipo via the tail vein. Representative images of tumor-bearing liver and other organs bioluminescent images examination. (D) The representative images of fluorescent signals derived from A/Lipo/Cy5-siRNA in the liver of C57BL/6 mice after the tail injection 1 hour. (D) Representative images and quantification analysis of immunohistochemistry staining for tumor YTHDF2 (Scale bar, 50 μm). (F) Western blotting analysis for YTHDF2 expression in different organs from mice treated with A/Lipo/siRNA. (G) The body weight of C57BL/6 mice was calculated at 4 weeks after implantation. Data are shown as the mean ± SD for (B). *, $p < 0.05$; **, $p < 0.01$; n.s., no significance.

Table S1. Clinicopathologic characteristics of patients with hepatocellular carcinoma in cohort 2.

Clinical characteristic	Number n=89	Percentage (%)
**Gender**		
Male	72	80.90
Female	17	19.10
**Age (years)**		
≤50	42	47.19
>50	47	52.81
**AFP (μg/L)**		
≤20	33	37.08
>20	56	62.92
**ALT (U/L)**		
≤75	86	96.63
>75	3	3.37
**HBV**		
Negative	6	6.74
Positive	83	93.26
**Cirrhosis**		
No	4	4.49
Yes	85	95.51
**Tumor size (cm)**		
≤5	45	50.56
>5	44	49.44
**Tumor number**		
Single	73	82.02
Multiple	16	17.98

**Tumor encapsulation**		
None	33	37.08
Complete	56	62.92
**Macrovascular invasion**		
No	78	87.64
Yes	11	12.36
**Microvascular invasion**		
No	61	68.54
Yes	28	31.46
**Satellite nodules**		
No	67	75.28
Yes	22	24.72
**Child-Pugh score**		
A	81	91.01
B	8	8.99
**BCLC stage**		
0+A	64	71.91
B+C	25	28.09

Table S2. Correlation between YTHDF2 expression and clinicopathologic characteristics in HCC patients in cohort 2.

**Clinical characteristic**	**Relative YTHDF2 expression**		***P* value**
	**Low (n=45)**	**High (n=44)**	
**Gender**			
Male	35	37	0.656
Female	10	7	

**Age (years)**			
≤50	20	22	0.755
>50	25	22	
**AFP (μg/L)**			
≤20	23	10	**0.011**
>20	22	34	
**ALT (U/L)**			
≤75	42	44	0.125*
>75	3	0	
**HBV**			
Negative	5	1	0.986*
Positive	40	43	
**Cirrhosis**			
No	2	7	0.073*
Yes	43	37	
**Tumor size (cm)**			
≤5	29	16	**0.015**
>5	16	28	
**Tumor number**			
Single	38	35	0.745
Multiple	7	9	
**Tumor encapsulation**			
None	14	22	0.109
Complete	31	22	
**Macrovascular invasion**			
No	42	35	0.997*
Yes	3	9	
**Microvascular invasion**			

No	34	27	0.225
Yes	11	17	
**Satellite nodules**			
No	36	31	0.425
Yes	9	13	
**Child-Pugh score**			
A	41	40	0.656*
B	4	4	
**BCLC stage**			
0+A	37	27	**0.029**
B+C	8	17	

Examined by Chi-square test. Bold number indicates *P* value<0.05; * indicates Fisher-exact test.

Table S3. Univariate and multivariate analysis of factors associated with survival of 89 HCC patients in cohort 2.

	**Survival**					
	**Univariate analysis**			**Multivariate analysis**		
	**HR**	**95%CI**	**P value**	**HR**	**95%CI**	**P value**
**Gender (male vs female)**	1.417	0.626-3.204	0.403			
**Age (>50 vs≤50)**	0.854	0.459-1.587	0.617			
**AFP (>20 vs≤ 20 μg/L)**	1.958	0.995-3.854	0.052			
**ALT (>75 vs ≤75 U/L)**	2.114	0.508-8.805	0.304			

**HBV (positive vs negative)**	3.739	0.901-15.511	0.069			
**Cirrhosis (yes vs no)**	1.035	0.368-2.909	0.948			
**Tumor size (>5 vs ≤5 cm)**	1.714	0.915-3.211	0.093			
**Tumor number (multiple vs single)**	2.707	1.372-5.340	**0.004**	0.927	0.232-3.702	0.915
**Tumor encapsulation (none vs complete)**	0.475	0.254-0.888	**0.020**	0.971	0.460-2.053	0.940
**Macrovascular invasion (yes vs no)**	3.987	1.878-8.463	**0.000**	2.756	0.865-8.787	0.086
**Microvascular invasion (yes vs no)**	2.161	1.150-4.061	**0.017**	1.034	0.491-2.177	0.929
**Child-Pugh score (B vs A)**	1.076	0.383-3.025	0.889			
**Satellite nodules (yes vs no)**	4.168	2.210-7.861	**0.000**	4.096	1.613-10.400	**0.003**
**BCLC stage (B+C vs 0+A)**	2.951	1.576-5.524	**0.001**	0.910	0.245-3.506	0.910
**YTHDF2 expression (high vs low)**	2.694	1.385-5.238	**0.003**	2.104	1.055-4.536	**0.035**

Table S4. Univariate and multivariate analysis of factors associated with recurrence of 89 HCC patients in cohort 2.

	**Recurrence**					
	**Univariate analysis**			**Multivariate analysis**		
	**HR**	**95%CI**	**P value**	**HR**	**95%CI**	**P value**
**Gender (male vs female)**	1.707	0.836-3.486	0.142			
**Age (>50 vs≤50)**	0.917	0.542-1.551	0.746			
**AFP (>20 vs≤20 μg/L)**	1.505	0.875-2.589	0.140			

**ALT (>75 vs ≤75 U/L)**	1.187	0.289-4.876	0.812			
**HBV (positive vs negative)**	1.490	0.674-3.291	0.324			
**Cirrhosis (yes vs no)**	1.029	0.410-2.579	0.952			
**Tumor size (>5 vs ≤5 cm)**	1.527	0.903-2.582	0.114			
**Tumor number (multiple vs single)**	3.718	2.019-6.844	**0.000**	1.095	0.294-4.104	0.889
**Tumor encapsulation (none vs complete)**	0.555	0.327-0.943	**0.030**	1.215	0.631-2.337	0.560
**Macrovascular invasion (yes vs no)**	2.553	1.281-5.089	**0.008**	0.695	0.219-2.211	0.538
**Microvascular invasion (yes vs no)**	2.626	1.537-4.484	**0.000**	2.022	1.130-3.619	**0.018**
**Child-Pugh score (B vs A)**	1.139	0.488-2.658	0.763			
**Satellite nodules (yes vs no)**	3.638	2.069-6.398	**0.000**	2.396	1.096-5.232	**0.028**
**BCLC stage (B+C vs 0+A)**	3.648	2.111-6.306	**0.000**	2.298	0.636-8.298	0.204
**YTHDF2 expression (low vs high)**	2.041	1.196-3.484	**0.009**	1.875	1.052-3.342	**0.033**

Table S8. Antibodies used in this study.

**Antigens**	**Manufacturers**	**Application**
YTHDF2	24744-1-AP, Proteintech, China	1:1000 for WB<br>1:200 for IHC<br>1:100 for RIP<br>1:100 for IP<br>1:100 for IF
GAPDH	60004-1-Ig, Proteintech, China	1:1000 for WB

CD31	ab28364, Abcam, USA	1:100 for IHC
m^6A	ab151230, Abcam, USA	1: 100 for MeRIP
ETV5	66657-1-Ig, Proteintech, China	1:1000 for WB
	ab102010, Abcam, USA	1:200 for IHC<br>1:200 for ChIP
Flag	F3165, Sigma, USA	1:1000 for WB<br>1:100 for IP
HA	H3663, Sigma, USA	1:1000 for WB<br>1:100 for IP
eIF3b	sc-137214, Santa Cruz Biotechnology, USA	1:100 for IF
	10319-1-AP, Proteintech, China	1:1000 for WB
	ab264230, Abcam, USA	1:100 for RIP<br>1:100 for IP
GST	PTM-5046, Jingjie, China	1:2000 for WB
His	SLAB2803, Smart-Lifesciences, China	1:1000 for WB
PD-L1	#13684, Cell Signaling Technology, USA	1:1000 for WB
VEGFA	ab1316, Abcam, USA	1:200 for IHC
	19003-1-AP, Proteintech, China	1:1000 for WB<br>1:200 for IHC
Ki67	#9129, Cell Signaling Technology, USA	1:800 for IHC
	#12202, Cell Signaling Technology, USA	1:800 for IHC
H3K4me3	A22146, Abclonal, China	1:1000 for WB<br>1:200 for ChIP
H3K27ac	A22264, Abclonal, China	1:1000 for WB<br>1:200 for ChIP
HRP-conjugated anti-rabbit IgG	111-035-003, Jackson ImmunoResearch, USA	1:2000 for WB

HRP-conjugated anti-mouse IgG	115-035-003, Jackson ImmunoResearch, USA	1:2000 for WB
Alexa Flour 488-conjugated anti-rabbit IgG	A0423, Beyotime Institute of Biotechnology, China	1:500 for IF
Alexa Flour 555-conjugated anti-mouse IgG	A0460, Beyotime Institute of Biotechnology, China	1:500 for IF

Table S9. Primers used in this study.

Primers	Sequences (5'-3')
GAPDH-H-F	AAGGTGAAGGTCGGAGTCA
GAPDH-H-R	GGAAGATGGTGATGGGATTT
GAPDH-M-F	TGGATTTGGACGCATTGGTC
GAPDH-M-R	TTTGCACTGGTACGTGTTGAT
YTHDF2-H-F	TAGCCAACTGCGACACATTC
YTHDF2-H-R	CACGACCTTGACGTTCCTTT
YTHDF2-M-F	CAGGCAAGGCCGAATAATGC
YTHDF2-M-R	TCTCCGTTGCTCAGTTGTCC
ETV5-H-F	TCAGCAAGTCCCTTTTATGGTC
ETV5-H-R	GCTCTTCAGAATCGTGAGCCA
ETV5-M-F	GCCTTCCCTGCAGGCTTTTA
ETV5-M-R	AAGCAGCCCTTCGAGTTCAA
FOXC1-F	GGCGAGCAGAGCTACTACC
FOXC1-R	TGCGAGTACACGCTCATGG

JUN-F	GTGCCGAAAAAGGAAGCTGG
JUN-R	CTGCGTTAGCATGAGTTGGC
KLF11-F	GCATGACAGCGAAAGGTCTAC
KLF11-R	GGGGTCTTATCCGCAACAGG
MEIS2-F	GTGAGCCAAGGAGCAGCATA
MEIS2-R	GGATCCCCATGTGTTGCTGA
PRDM1-F	GGCGAGCAGAGCTACTACC
PRDM1-R	GGGATGGGCTTAATGGTGTAGAA
PRDM4-F	TCCTCTGTGAGCAATGCCTTG
PRDM4-R	CCACACATCACCCCTCGAT
RUNX1-F	AACCCAGCATAGTGGTCAGC
RUNX1-R	GCACTGTGGGTACGAAGGAA
ZBTB6-F	AAACAACAACCAACACACTGACC
ZBTB6-R	ACAGCTCTGGTGACTTCATTTC
HPRT1-H-F	TTGCTTTCCTTGGTCAGGCA
HPRT1-H-R	ATCCAACACTTCGTGGGGTC
HRPT1-M-F	GTTGGGCTTACCTCACTGCT
HRPT1-M-R	TAATCACGACGCTGGGACTG
Fluc-F	GTGTCCGATTCAGTCATGCC
Fluc-R	CCAGCAGGGCAGATTGAATC
Rluc-F	AAGATCAAGGCCATCGTCCA
Rluc-R	ACTCCTCAGGCTCCAGTTTC
PD-L1-H-F	TGGCATTTGCTGAACGCATTT
PD-L1-H-R	TGCAGCCAGGTCTAATTGTTTT
PD-L1-M-F	GCTCCAAAGGACTTGTACGTG
PD-L1-M-R	TGATCTGAAGGGCAGCATTTC
VEGFA-H-F	AGGGCAGAATCATCACGAAGT
VEGFA-H-R	AGGGTCTCGATTGGATGGCA

VEGFA-M-F	TATTCAGCGGACTCACCAGC
VEGFA-M-R	TATTCAGCGGACTCACCAGC
RCN2-F	CATCATGGAGCTGTCTGCCA
RCN2-R	AGGGCATTGCACAAGAGGAG
YTHDF2-promoter-ChIP-1-F	GCCTCCTGTGGTCAAGTGAT
YTHDF2-promoter-ChIP-1-R	CTCACGCCTGTAATCCCAGT
YTHDF2-promoter-ChIP-2-F	CTCCCAAAGTGCTGGGATTA
YTHDF2-promoter-ChIP-2-R	GTAAGGTCAGGCACCAAAGG
YTHDF2-promoter-ChIP-3-F	TTTTACCGAGTCCACCCAAG
YTHDF2-promoter-ChIP-3-R	TAGGGGAGATGCAAAGGATG
YTHDF2-promoter-ChIP-4-F	TTTCCTTGCTTCCCTCTTGA
YTHDF2-promoter-ChIP-4-R	CCCAGAGAGCAAAACACACA
PD-L1-promoter -ChIP-1-F	GGTAGACCCTGAACACTGCT
PD-L1-promoter-ChIP-1-R	AGCCATCTACCACTAACTAGCT
PD-L1-promoter-ChIP-2-F	AAGAAAAGGGAGCACACAGG
PD-L1-promoter-ChIP-2-R	AATGGGCCCAAGATGACAGA
PD-L1-promoter-ChIP-3-F	GGGACCCTGAGCATTCTTAAA
PD-L1-promoter-ChIP-3-R	TGTGTGTGTGTGTATGGGTG
PD-L1-promoter-ChIP-4-F	ACAGCTTTATTCCTAGGACACCA
PD-L1-promoter-ChIP-4-R	CCAAGGCAGCAAATCCAGTT
PD-L1-promoter-ChIP-Distant region-F	AGAGCTGAAAGGATCCCAGG
PD-L1-promoter-ChIP-Distant region-R	AGAGGGGCAGATTTCAGCTT
VEGFA-promoter-ChIP-1-F	TGGCTTCCCTTCCATATCCC
VEGFA-promoter-ChIP-1-R	AGTCCTGTCTCCACCACTTG
VEGFA-promoter-ChIP-2-F	CCACCAAACCACAGCAACAT
VEGFA-promoter-ChIP-2-R	AGGCATGGACTGAGAATGGG
VEGFA-promoter-ChIP-3-F	CCAACAGGTCCTCTTCCCTC
VEGFA-promoter-ChIP-3-R	CCAGGGGAGAAGAATTTGGC

VEGFA-promoter-ChIP-4-F	CGCACGTAACCTCACTTTCC
VEGFA-promoter-ChIP-4-R	CAATGAAGGGGAAGCTCGAC
VEGFA-promoter-ChIP-Distant region-F	ATTCCCTGTAACCCTGCCTC
VEGFA-promoter-ChIP-Distant region-R	TCCCCTCCCTTAATCCTCCA
Alb-Cre-F	GAAGCAGAAGCTTAGGAAGATGG
Alb-Cre-R	TTGGCCCCTTACCATAACTG
LoxP site1-F	GGGTGGAATGCATTTTGAGAAGC
LoxP site1-R	AAAGGTCAGTGTATTGGCTCCTT
LoxP site2-F	TGTGCATAGCTCTTTTGGCTAATG
LoxP site2-R	TACACTAGAAGGACACACTGATGC
Ythdf2^{LKO} Loxp-F	GGTCAAGGAAACAAAGGTAAGACC
Ythdf2^{LKO} Loxp-R	GGCTGTCCTAAGAGGAGACAATAAA

Table S10. Antibodies used in flow cytometric analysis.

Antibodies	Source
Fixable viability Stain 700	BD, 564406
anti-CD45-mice	BD, 557659
anti-CD3-mice	BD, 562600
anti-CD8-mice	BD, 553030
anti-CD4-mice	BD, 563151
anti-Granzyme B-mice	BD, 130-116-486
anti-CD11b-mice	BD, 557396
anti-CD11c-mice	BD, 566504
anti-F4/80-mice	BD, 565411
anti-Ly6G-mice	BD, 560601
anti-CD31-mice	BD, 551262
anti-PD-L1-mice	BD, 558091

anti-PD-L1-human	BD, 568721

Table S11. Oligonucleotides for knocking down the indicated genes.

Targets	Sequences (5'-3')
**RNAi**	
NC	sense: UUCUCCGAACGUGUCACGUTT
si1-	sense: GGAGAGAAAUUCAAGCAAATT
si2-	sense: GUGGGAUAUUCCAGAGAAATT
si-	sense: GCAAACTTGCAGTTTATGTAT
si1-	sense: CCUUCUACCGGGACAACAATT
si2-	sense: GGGAAUAGYAGCUGUCAAATT
si1-JUN	sense: GGAUCAAGGCGGAGAGGAATT
si2-JUN	sense: CAGCUUAAACAGAAAGUCATT
si1-	sense: UGGACAAAGUAGCAUGUUATT
si2-	sense: CUGGGAUGGCUGUGAUAAATT
si1-	sense: GAUACAAGCAAUACAAGUATT
si2-	sense: CCGAAGAGCAGAAGAAACATT
si1-	sense: CAAUGGAAAUGUUGGAUUATT
si2-	sense: GCAAAGAAAGGAAGUGGAATT
si1-	sense: UUAAAGAAGAGGAAAGCAATT
si2-	sense: UGGCAGAAGCAGUGGUGAATT
**shRNAs**	
sh1-YTDFH	CCGGTACTGATTAAGTCAGGATTAACTCGAGTTAATCCTGACTTAATCAGTATTTTTG
sh2-YTDFH	CCGGGCTACTCTGAGGACGATATTCCTCGAGGAATATCGTCCTCAGAGTAGCTTTTTG

sh1-ETV5-h	GATCCGTCCATGGCTTTCCCGGATAACCTCGAGGTTATCCGGGAAAGCCATGGATTTTTTG
sh2-ETV5-h	GATCCGCACCTCCAACCAAGATCAAACCTCGAGGTTTGATCTTGGTTGGAGGTGTTTTTTG
sh1-ETV5-	GATCCGCGACCTTTGATTGACAGAAACTCGAGTTTCTGTCAATCAAAGGTCGCTTTTTTG
sh2-ETV5-	CACAGATCTCGAGATCTGTGTCCACAAACTTCCTTTTTTG
sh-PD-L1-m	CCGGGGAGAAATGTGGCGTTGAAGACTCGAGTCTTCAACGCCACATTTCTCCTTTTTG
sh-VEGFA-m	CCGGGCCAGCACATAGGAGAGATGACTCGAGTCATCTCTCCTATGTGCTGGCTTTTTG

References:

[1] Q. Gao, H. Zhu, L. Dong, W. Shi, R. Chen, Z. Song, C. Huang, J. Li, X. Dong, Y. Zhou, Q. Liu, L. Ma, X. Wang, J. Zhou, Y. Liu, E. Boja, A. I. Robles, W. Ma, P. Wang, Y. Li, L. Ding, B. Wen, B. Zhang, H. Rodriguez, D. Gao, H. Zhou, J. Fan, *Cell* **2019**, *179* (5), 1240, https://doi.org/10.1016/j.cell.2019.10.038.

[2] C. H. Mermel, S. E. Schumacher, B. Hill, M. L. Meyerson, R. Beroukhim, G. Getz, *Genome Biol* **2011**, *12* (4), R41, https://doi.org/10.1186/gb-2011-12-4-r41.

[3] G. Q. Zhu, Z. Tang, R. Huang, W. F. Qu, Y. Fang, R. Yang, C. Y. Tao, J. Gao, X. L. Wu, H. X. Sun, Y. F. Zhou, S. S. Song, Z. B. Ding, Z. Dai, J. Zhou, D. Ye, D. J. Wu, W. R. Liu, J. Fan, Y. H. Shi, *Cell Discov* **2023**, *9* (1), 25, https://doi.org/10.1038/s41421-023-00529-z.

[4] R. Satija, J. A. Farrell, D. Gennert, A. F. Schier, A. Regev, *Nat Biotechnol* **2015**, *33* (5), 495, https://doi.org/10.1038/nbt.3192.

[5] M. Efremova, M. Vento-Tormo, S. A. Teichmann, R. Vento-Tormo, *Nat Protoc* **2020**, *15* (4), 1484, https://doi.org/10.1038/s41596-020-0292-x.

[6] Z. Huang, L. Chu, J. Liang, X. Tan, Y. Wang, J. Wen, J. Chen, Y. Wu, S. Liu, J. Liao, R. Hou, Z. Ding, Z. Zhang, H. Liang, S. Song, C. Yang, J. Zhang, T. Guo, X. Chen, B. Zhang, *Hepatology* **2021**, *74* (1), 214, https://doi.org/10.1002/hep.31673.

[7] J. Wen, Z. Huang, Y. Wei, L. Xue, Y. Wang, J. Liao, J. Liang, X. Chen, L. Chu, B. Zhang, *Cell Mol Biol Lett* **2022**, *27* (1), 79, https://doi.org/10.1186/s11658-022-00370-4.

[8] L. Zhong, D. Liao, M. Zhang, C. Zeng, X. Li, R. Zhang, H. Ma, T. Kang, *Cancer Lett* **2019**, *442*, 252, https://doi.org/10.1016/j.canlet.2018.11.006.

[9] S. Han, L. Xue, Y. Wei, T. Yong, W. Jia, Y. Qi, Y. Luo, J. Liang, J. Wen, N. Bie, H. Liang, Q. Liu, Z. Ding, X. Yang, L. Gan, Z. Huang, X. Chen, B. Zhang, *Adv Sci (Weinh)* **2023**, e2207080, https://doi.org/10.1002/advs.202207080.
